# Delta-secretase cleaves amyloid precursor protein and regulates the pathogenesis in Alzheimer's disease

**DOI:** 10.1038/ncomms9762

**Published:** 2015-11-09

**Authors:** Zhentao Zhang, Mingke Song, Xia Liu, Seong Su Kang, Duc M. Duong, Nicholas T. Seyfried, Xuebing Cao, Liming Cheng, Yi E. Sun, Shan Ping Yu, Jianping Jia, Allan I. Levey, Keqiang Ye

**Affiliations:** 1 Department of Pathology and Laboratory Medicine, Emory University School of Medicine, Atlanta, Georgia 30322, USA; 2 Department of Neurology, Renmin Hospital of Wuhan University, Wuhan 430060, China; 3 Department of Anesthesiology, Emory University School of Medicine, Atlanta, Georgia 30322, USA; 4 Department of Neurology, Center for Neurodegenerative Diseases, Emory University School of Medicine, Atlanta, Georgia 30322, USA; 5 Department of Biochemistry, Center for Neurodegenerative Diseases, Emory University School of Medicine, Atlanta, Georgia 30322, USA; 6 Department of Neurology, Union Hospital, Tongji Medical College, Huazhong University of Science and Technology, Wuhan 430022, China; 7 Translational Center for Stem Cell Research, Tongji Hospital, Department of Regenerative Medicine, Tongji University School of Medicine, Shanghai 200065, China; 8 Department of Psychiatry and Biobehavioral Sciences, UCLA School of Medicine, Los Angeles, California 90095, USA; 9 Department of Neurology, Xuan Wu Hospital of Capital Medical University, 45 Changchun Street, Beijing 100053, China

## Abstract

The age-dependent deposition of amyloid-β peptides, derived from amyloid precursor protein (APP), is a neuropathological hallmark of Alzheimer's disease (AD). Despite age being the greatest risk factor for AD, the molecular mechanisms linking ageing to APP processing are unknown. Here we show that asparagine endopeptidase (AEP), a pH-controlled cysteine proteinase, is activated during ageing and mediates APP proteolytic processing. AEP cleaves APP at N373 and N585 residues, selectively influencing the amyloidogenic fragmentation of APP. AEP is activated in normal mice in an age-dependent manner, and is strongly activated in 5XFAD transgenic mouse model and human AD brains. Deletion of AEP from 5XFAD or APP/PS1 mice decreases senile plaque formation, ameliorates synapse loss, elevates long-term potentiation and protects memory. Blockade of APP cleavage by AEP in mice alleviates pathological and behavioural deficits. Thus, AEP acts as a δ-secretase, contributing to the age-dependent pathogenic mechanisms in AD.

## Abstract (web summary)

Age is the greatest risk factor for Alzheimer's disease, yet how ageing regulates disease pathology is unclear. Here, the authors find that asparagine endopeptidase expression increases with age and cleaves APP, contributing to ß-amyloid production and cognitive defects in a transgenic mouse model.

## Graphical abstract

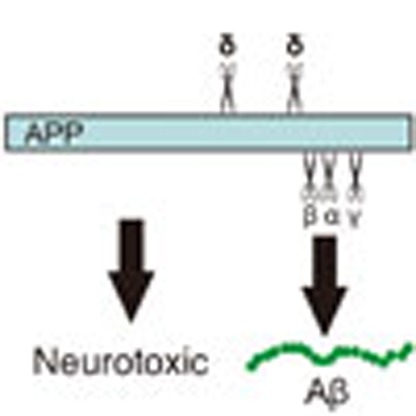

Amyloid β (Aβ) is a key pathogenic factor in Alzheimer's disease (AD). It aggregates to form neurotoxic oligomers and insoluble deposits in plaques in the brains of affected individuals, contributing to the progressive loss of synaptic function and cognitive impairment. Aβ peptides are derived from the sequential proteolytic processing of amyloid precursor protein (APP) by a group of proteases^1^,^2^. APP is a type I oriented membrane protein with its amino terminus within the lumen/extracellular space and its carboxyl terminus within the cytosol^3^,^4^. Three proteases, α-, β- and γ-secretases, regulate APP processing^2^. Commitment of APP into amyloidogenic and non-amyloidogenic processing depends on the cellular levels of α- and β-secretases and the traffic of APP to subcellular organelles expressing these proteases. The amyloidogenic pathway leads to Aβ generation, while the anti-amyloidogenic pathway prevents Aβ generation^5^.

β-Secretase activity mediates the initial and rate-limiting processing step leading to Aβ generation^6^. β-Secretase (BACE1) is a membrane-bound aspartyl protease with its active site in the lumen/extracellular space^7^ and an acidic pH optimum (around pH 4.5; ref. 8), consistent with the major site of β-secretase activity within endosomes. The β-secretase-cleaved C-terminal portion of APP is subsequently processed by γ-secretase to liberate Aβ. Mounting evidence shows that γ-secretase cleaves APP in the endosomal/lysosomal system, including phagosomes and autophagosomes^9^. The protease activity of γ-secretase is also regulated by pH with optimal activity at pH 6.3 (ref. 10). An alternative pathway of anti-amyloidogenic processing of APP occurs within the Aβ domain between residues K612 and L613 in APP^11^,^12^ and results in the secretion of the large APP amino-terminal domain and the generation of α-CTF (C83). This cleavage is performed by α-secretases, which in neurons includes ADAM10 (ref. 13). α-Secretase processing of APP occurs in the *trans*-Golgi network and the plasma membrane^14^. On the basis of the relative BACE1 expression levels, neurons have been suggested to be the major source of Aβ produced in the brain^5^.

Mammalian asparagine endopeptidase (AEP), also known as legumain, is a widely distributed lysosomal cysteine protease that cleaves after asparagine residues^15^,^16^. AEP activation is autocatalytic and requires sequential removal of C- and N-terminal propeptides at different pH thresholds^17^. Disruption of AEP leads to late endosome and lysosome abnormalities with accumulation of electron-dense and/or membranous materials^18^. AEP has diverse physiological functions in the brain. Neuronal AEP is activated by acidosis during excitotoxicity and contributes to neuronal apoptosis by degrading the DNase inhibitor SET, a binding partner of APP^19^,^20^. AEP cleavage of SET (I_2_^PP2A^) inhibits PP2A and upregulates tau hyperphosphorylation^21^. AEP also cleaves TDP-43 in human cases of frontotemporal lobar degeneration^22^. Moreover, AEP cleaves tau, mediating neurofibrillary pathology in AD^23^. AEP is inhibited by cystatin C, a secreted protein found in human cerebrospinal fluid (CSF) that binds soluble Aβ and inhibits its oligomerization, and which is reduced in AD^24^,^25^. Cystatin C interacts with active AEP in the lysosome and acts as an endogenous inhibitor of AEP^26^,^27^. Together, these observations led us to hypothesize that AEP plays an important role in AD pathogenesis.

In this report, we show that APP is a physiological substrate of AEP. AEP increases with ageing and proteolytically cleaves APP in the ectodomain, affecting the rate of BACE1 cleavage of the resultant substrate. Knockout of AEP *in vivo* using 5XFAD and APP/PS1 mouse models of AD reduces Aβ burden, improves synapse integrity and preserves memory. Blockade of APP cleavage by AEP prevents pathological and behavioural defects induced by overexpression of APP. These findings suggest that AEP is activated during ageing and promotes Aβ production, contributing to AD onset and progression. Hence, AEP may act as a novel δ-secretase that mediates APP fragmentation and amyloidogenesis.

## Results

### APP is a physiological substrate of AEP

**Figure 1:**
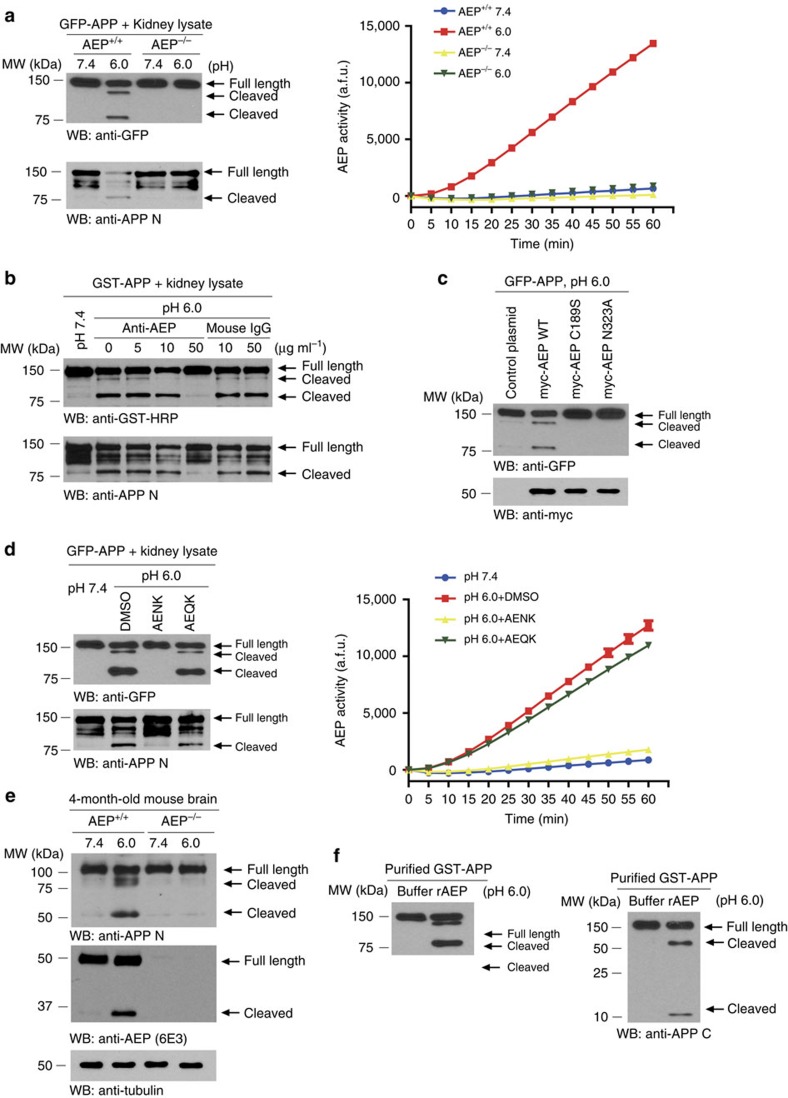
AEP cleaves APP *in vitro*. (**a**) APP cleavage by AEP^+/+^ kidney lysates. APP was cleaved at pH 6.0 by AEP^+/+^ kidney lysates (left panels). The enzymatic activity of AEP was determined using AEP activity assay (right panel; mean±s.e.m. of three independent experiments). (**b**) Antibody titration assay. Kidney lysates were incubated with AEP-specific antibody for 5 min, and then incubated with GST-APP for 30 min. The processing of APP was determined using western blot. (**c**) Mutants of AEP C189 and N323 diminish APP cleavage. (**d**) Blocked of APP cleavage by Fmoc-Ala-Glu-Asn-Lys-NH2 (AENK) peptide (left panel). AEP activity was inhibited by AENK but not by Fmoc-Ala-Glu-Gln-Lys-NH2 (AEQK) (right panel; mean±s.e.m.; *n*=3). (**e**) Cleavage of endogenous APP by AEP. (**f**) Western blot showing the processing of purified GST-APP by recombinant AEP. The AEP-derived APP fragments were detected using anti-APP N-terminal antibody (left panel) and anti-APP C-terminal antibody (right panel). a.f.u., arbitrary fluorescence unit; DMSO, dimethylsulfoxide; MW, molecular weight; WB, western blot.

To investigate whether APP is a substrate of AEP, we prepared kidney lysates derived from wild-type (WT; +/+) or AEP knockout (−/−) mice at different pH to inactivate (pH 7.4) or activate AEP (pH 6.0), respectively, and incubated with recombinant green fluorescent protein (GFP)-APP. Immunoblotting analysis revealed robust APP cleavage with two major fragments in WT samples under pH 6.0 but not pH 7.4. By contrast, we failed to observe any APP cleavage in AEP-null samples regardless of pH values. We also validated the APP fragmentation with anti-APP N-terminal antibody (Fig. 1a, left panels), and confirmed the pH dependence of AEP enzyme activity in WT samples (Fig. 1a, right panel). We used inhibitory anti-AEP antibodies to verify the role of AEP. As anti-AEP doses gradually increased, APP fragmentation was progressively inhibited and completely blocked at 50 μg ml^−1^. In contrast, the same concentrations of anti-mouse IgG failed to block APP cleavage (Fig. 1b). Mutation of either of two key residues in AEP blunted its proteolytic activity against APP (Fig. 1c), including C189 that is essential for the cysteine proteinase activity and N323 that plays a critical role in the cleavage and maturation of AEP^17^,^19^. In addition, the selective AEP inhibitor peptide AENK suppressed APP cleavage, while an inactive peptide AEQK had no effect^28^. AEP enzymatic activity tightly correlated with the APP shedding pattern (Fig. 1d). We also explored whether or not endogenous APP in mouse brain can be processed by AEP. Endogenous APP was robustly cleaved in WT (AEP^+/+^) mouse brain lysates with acidic conditions that activate AEP, whereas the cleavage was abrogated at pH 7.4 where AEP is catalytically inactive. APP cleavage was also absent in brain lysates from AEP knockout mice (AEP^−/−^; Fig. 1e). To ascertain if APP is indeed a direct substrate of AEP, we incubated purified glutathione S-transferases (GST)-APP with active recombinant human AEP. AEP potently cleaved GST-APP recombinant proteins with the same pattern as in the tissues (Fig. 1f). Thus, the biochemical cleavage assay, antibody or peptide inhibition assay and studies with AEP knockout tissues all indicate that APP is a physiological substrate of AEP.

### AEP cleaves APP at N373 and N585 residues

**Figure 2:**
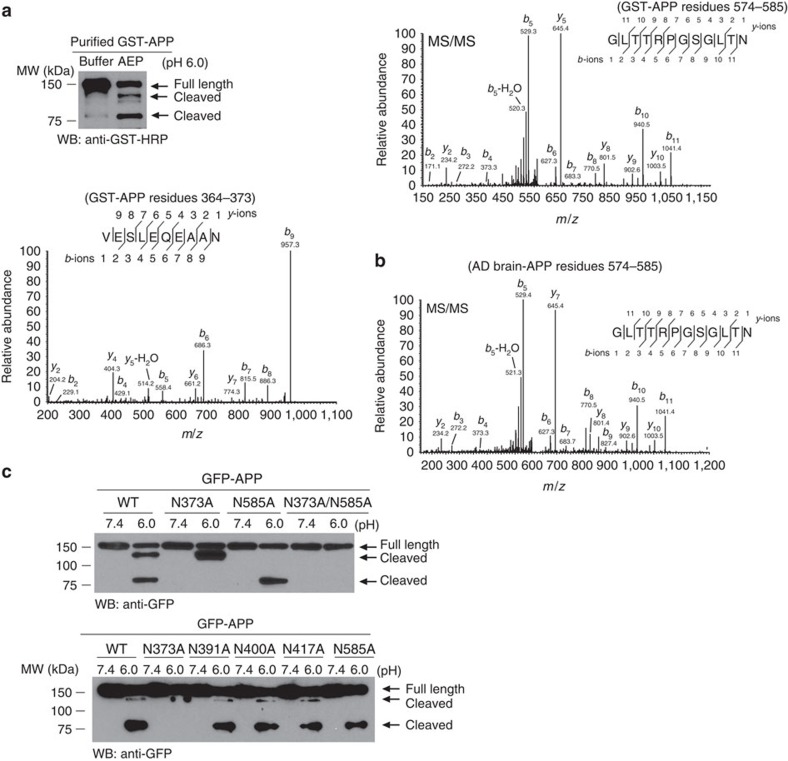
AEP cleaves APP at N373 and N585 residues. (**a**) Proteomic analysis of APP recombinant proteins processed by AEP. The detected peptide sequences indicate that N585 and N373 are the two main cleavage sites with the shed bands of molecular weight (MW) ∼130 and 80 kDa, respectively. (**b**) Proteomic analysis of APP fragments in AD brain. APP fragments cleaved after N585 was detected. (**c**) Processing of various mutant APP by AEP.

To identify potential AEP cleavage sites on APP, we generated a series of truncated APP proteins with sequential deletions of N-terminal domains (Supplementary Fig. 1) and tested them in the biochemical cleavage assay. Deletion of the N terminus proximal to amino acid 289 did not affect APP cleavage (Supplementary Fig. 1), indicating that AEP cleaves APP after residue 289. Next, we purified the cleaved recombinant protein products and analysed them by mass spectrometry (MS), identifying two main cleavage sites at N585 and N373 that yield fragments of molecular weight ∼130 and 80 kDa, respectively (Fig. 2a). The amino acid number is based on the APP695 isoform. To determine if the same APP cleavages occur in human brain from AD cases, we immunoprecipitated brain lysates with anti-APP N-terminal antibody and subjected the samples to proteomic analysis. An APP fragment terminated at N585 was detected (Fig. 2b). Mutations of N373A or N585A abolished the 80- or 130-kDa fragments, respectively, and the APP double mutant (N373A/N585A) was resistant to both cleavages. By contrast, mutations at other sites (N391A, N400A or N417A) had no effect on APP fragmentation (Fig. 2c). Thus, these studies suggest that N373 and N585 are the two major AEP cleavage sites in APP.

### AEP regulates Aβ production

**Figure 3:**
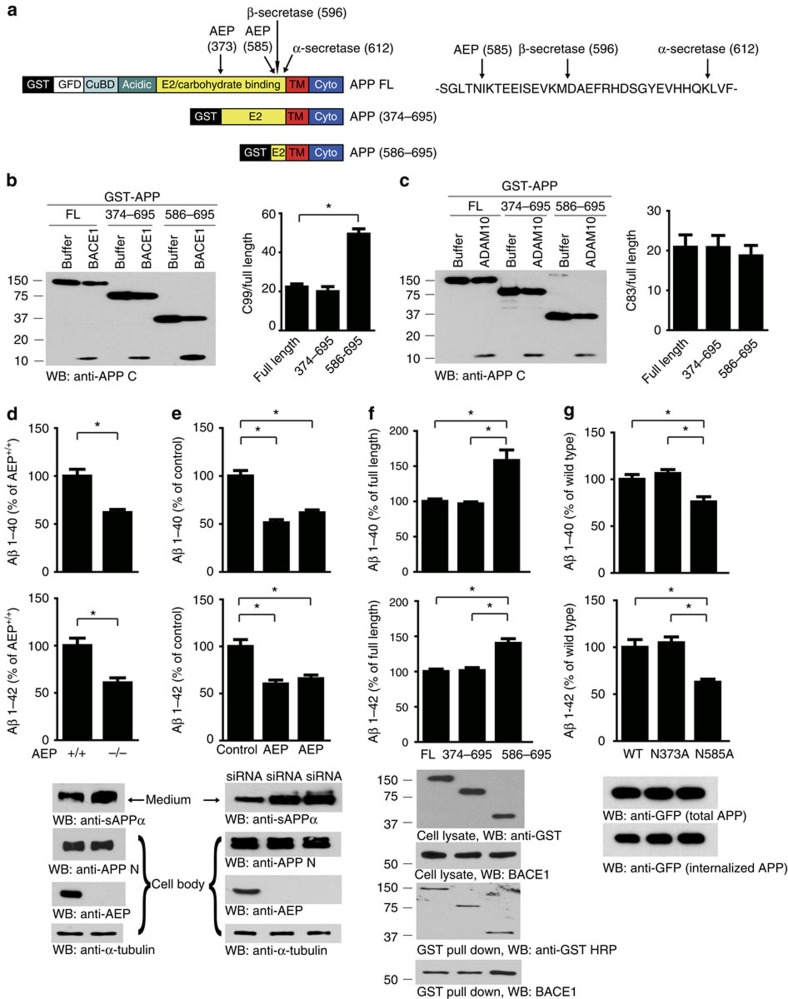
Cleavage of APP by AEP at N585 accelerates Aβ production. (**a**) Schematic drawing of APP cleavage by AEP, β-secretase and α-secretase. (**b**,**c**) Western blot (WB) showing the processing of APP fragments by α- and β-secretase. Arrows indicate APP C-terminal fragments generated by BACE1 (C99, **b**) and ADAM10 (C83, **c**). The percentage of C99 and C83 was quantified. (**d**–**g)** ELISA results showing the concentrations of Aβ 1–40 and Aβ 1–42 in medium from AEP^+/+^ and AEP^−/−^ primary neurons (**d**), HEK293 cells stably transfected with APP (**e**), HEK293 cells transiently transfected with GST-tagged APP fragments (**f**) or GFP-tagged mutant APP (**g**). The binding of APP fragments with BACE1 was analysed using GST pull-down assay (**f**). The internalized APP was assessed by internalization assay (**g**). Data are mean±s.e.m. of three independent experiments, and were analysed using one-way analysis of variance followed by *post hoc* comparison, *P<0.01.

APP can be processed by several proteases, with both α- and β-secretases shedding APP ectodomains (Fig. 3a). To determine if AEP processing of APP affects α- and β-secretase cleavages, recombinant APP proteins corresponding to the two AEP-processed derivatives (APP_374–695_ and APP_586–695_) were tested as substrates for ADAM10 and BACE1 protease, respectively. BACE1 processed both full-length APP and the truncated APP_374–695_ fragment at the same rate. Interestingly, BACE1 processing of the APP_586–695_ fragment yielded more C99 product than full-length APP or APP_374–695_ (Fig. 3b). By contrast, ADAM10 processed all these proteins at the same rate (Fig. 3c), indicating that APP_586–695_ might be a better substrate for BACE1 than full-length APP or APP_374–695_. To further test this possibility, we incubated purified GST-APP_586–695_ with BACE1 in the presence or absence of AEP-generated APP N-terminal fragment APP_1–373_ or APP_1–585_, respectively. APP_1–585_, but not APP_1–373_ decreased C99 production in a concentration-dependent manner (Supplementary Fig. 2a). However, AEP-generated APP N-terminal fragments had no effect on ADAM10 cleavage of APP_586–695_ (Supplementary Fig. 2b). These data suggest that the N terminus of APP_1–585_ might inhibit BACE1 processing of the resultant cleavage product APP_586–695_. Conceivably, cleavage of APP by AEP at N585 may relieve the steric hindrance of the N terminus and thereby accelerate APP processing by BACE1. Since BACE1 is the rate-limiting enzyme for Aβ production, we also explored the role of AEP in Aβ production in neurons. Remarkably, while total APP protein levels were similar between WT and AEP^−/−^ neurons, the concentrations of Aβ40 and Aβ42 in the conditioned medium of AEP^−/−^cultures were significantly lower than those in AEP^+/+^ cultures. The concentration of sAPPα in the AEP^−/−^ conditioned medium was higher than that of AEP^+/+^ cultures (Fig. 3d). Though AEP is assumed to be highly expressed in microglia^29^, we found it is abundant in primary neuronal cultures as well. The percentage of neurons was about 96% in both AEP^+/+^ and AEP^−/−^ neuronal cultures (Supplementary Fig. 2c). To further confirm these results, we transfected HEK293 cells stably expressing human APP with two different AEP siRNAs or control siRNA. Depletion of AEP in these cells significantly reduced Aβ production and increased sAPPα levels without altering the level of total APP (Fig. 3e). Conversely, overexpression of the APP_586–695_ fragment markedly elevated Aβ production compared with overexpression of full-length APP and the APP_374–695_ fragment. To further evaluate the substrate preference of BACE1 for the various forms of APP, we also carried out GST pull-down assays, and found that the APP_586–695_ fragment bound more BACE1 than full-length APP and APP_374–695_ (Fig. 3f). Furthermore, when the APP_596–695_ fragment was co-expressed in HEK293 cells with increasing amounts of the myc-APP_1–585_ or myc-APP_1–373_, the longer APP_1–585_ fragment selectively decreased the production of Aβ in a dose-dependent manner and reduced the binding of BACE1 to APP_586–695_ (Supplementary Fig. 2d). Accordingly, APP mutation analysis revealed Aβ production was reduced by inhibition of AEP cleavage of APP at N585, but not at N373. Since cell surface trafficking of APP affects its processing, we tested the possibility that these mutations affect the internalization of cell surface APP. Cell surface biotinylation assays demonstrated that neither the N585 nor the N373 mutations had any effect on APP internalization (Fig. 3g). Hence, AEP cleavage of APP at N585 facilitates the subsequent processing by BACE1 and generation of Aβ.

Since AEP pre-processing of APP directly influences subsequent cleavage by BACE1, we sought to determine if other proteases might affect AEP cleavage of APP. APP is processed by caspase-3 and cathepsin in addition to the secretases^30^,^31^. We employed pharmacological inhibitors of these proteases and found that only the AEP inhibitory peptide AENK antagonized APP processing by AEP, whereas other small molecular inhibitors and inactive peptide AEQK were without effect (Supplementary Fig. 3a). Moreover, APP point mutants that influence cleavage by a variety of secretases were strongly cleaved by AEP (Supplementary Fig. 3b), suggesting that APP cleavage by secretases does not affect AEP processing of its substrate APP. To further define the role of BACE1, γ-secretase or α-secretase in AEP processing of APP, we also used BACE1^−/−^, PS1/PS2^−/−^ and ADAM10/17^−/−^ mouse embryonic fibroblast cells. Proteolytic analysis revealed that AEP initiated APP fragmentation in a time-dependent manner with comparable rates regardless of the presence or absence of BACE1, γ-secretase or α-secretase (Supplementary Fig. 3c). Therefore, these studies demonstrate that the secretases do not interfere with AEP's cleavage of APP.

### AEP interacts with APP in the endolysosomal organelles

**Figure 4:**
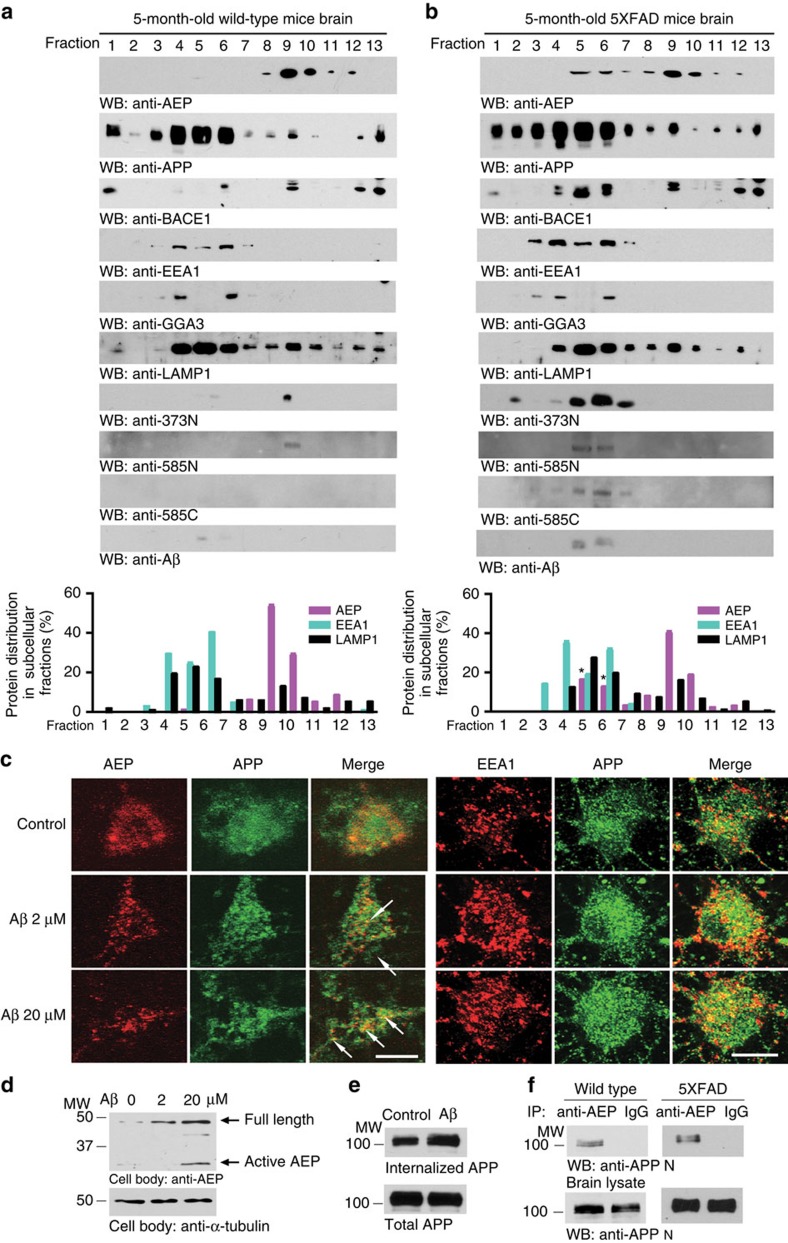
AEP interacts with APP in the endolysosomal system. (**a**,**b**) AEP and APP distribution in the subcellular fractions. Brain samples from 5-month-old WT (**a**) and age-matched 5XFAD (**b**) mice were homogenated and fractionated on a discontinuous sucrose gradient. The fractions were analysed by western blotting (WB) for AEP, APP fragments, BACE1, EEA1 (endosome marker), GGA3 (*trans*-Golgi network maker) and LAMP1 (lysosome marker). The relative amount of AEP, EEA1 and LAMP1 in each fraction was quantified (mean±s.e.m. of three independent experiments, *t*-test, *P<0.01 compared with WT mice). (**c**) APP co-localizes with AEP and EEA1. Primary neuronal cultures (DIV 12) were treated with 2 or 20 μM of pre-aggregated Aβ for 24 h, followed by immunostaining with various antibodies including anti-AEP, anti-APP or anti-EEA1. Aβ treatment increased the co-localization of APP and AEP in primary cortical neurons (left panels). APP co-localized with the endosomal marker EEA1 as well (right panels). Shown are the representative figures of two independent experiments. Scale bar, 10 μm. (**d**) Aβ treatment elicits AEP activation in neurons in a dose-dependent manner. (**e**) Internalization assay showing the effect of 20 μM Aβ on APP endocytosis. (**f**) Co-immunoprecipitation of APP and AEP in WT and 5XFAD mouse brain. AEP in mouse brain lysates was immunoprecipitated with anti-AEP antibody and analysed by immunoblotting with anti-APP antibody. MW, molecular weight.

To explore how a lysosomal protease AEP cleaves APP and leads to secretion of the relevant APP fragments, we investigated the subcellular basis for the interaction between AEP and APP in brain. Brain samples from WT, 5XFAD and tau P301S mice were fractionated by differential centrifugation on a sucrose discontinuous gradient to separate cellular organelles. For the WT brain samples, AEP was enriched in fractions 9 and 10, containing the specific lysosomal marker LAMP1, and APP was enriched in fractions 4–6, co-enriched with EEA1, a specific marker for the endosome, and GGA3, a Golgi-localized adaptor protein involved in BACE1 trafficking^32^ (Fig. 4a). In the aged WT mouse brain, 5XFAD and tau P301S transgenic mouse brain, AEP distribution was more widely distributed in fractions 5–12, consistent with its upregulation during ageing and in AD (Fig. 6). Moreover, AEP overlapped with APP in fractions 5 and 6 together with the endolysosomal markers LAMP1 and EEA1. BACE1 was highly enriched in the same fractions (Fig. 4b and Supplementary Fig. 4a,b). To detect the APP fragments derived from AEP processing in the fractionated brain samples, we developed cleavage-site-specific antibodies, which selectively bind the following AEP-derived fragments: APP_1–373_ (anti-APP 373N), APP_1–585_ (anti-APP 585N) and APP_586–695_ (anti-APP 585C). The specificity of these antibodies was confirmed by western blot and immunohistochemistry (Supplementary Fig. 5a–e). APP_1–373_ and APP_1–585_ fragments were enriched in fractions 5 and 6 of 5XFAD mice. Thus, these data suggest that AEP cleaves APP in the endolysosomal system. Accordingly, the cleavage of APP by AEP in endolysosomes may promote the subsequent BACE1 cleavage and production of Aβ.

Aβ exerts a variety of deleterious effects on cells including altering membrane protein traffic^33^. To explore the effects of Aβ on the membranous compartments where AEP may cleave APP, we treated primary cortical neurons with different doses of pre-aggregated Aβ for 24 h and determined the subcellular distribution of APP and AEP by immunofluorescent staining. Remarkably, Aβ (20 μM) treatment triggered the co-localization of APP and AEP in primary neurons (Fig. 4c, left panels and Supplementary Fig. 6a). APP also co-localized with the endosomal marker EEA1 (Fig. 4c, right panels and Supplementary Fig. 6b). The co-localization of AEP with EEA1 and LAMP1 in Aβ-treated neurons and 5XFAD mice brain was confirmed by confocal microscopy (Supplementary Fig. 6c–f). Notably, Aβ treatment elicited AEP expression and activation (Fig. 4d). Using an internalization assay, we found that Aβ treatment promoted the endocytosis of APP (Fig. 4e). Moreover, APP and AEP were co-immunoprecipitated from both WT and 5XFAD brain lysates, confirming their interaction (Fig. 4f).

AEP is secreted extracellularly in the tumour microenvironment and associated with cell surface^26^. We found that AEP was secreted into the CSF, and interestingly, the AEP-cleaved APP_1–585_ fragment was significantly higher in human AD CSF than in healthy controls (Supplementary Fig. 7a,b). Furthermore, exogenous His-tagged AEP recombinant proteins bound to APP on the cell surface, and were internalized and subsequently processed to the active form (Supplementary Fig. 7c,d). Thus, AEP interacts with APP on the cell surface, and the complex may be endocytosed. To study how Aβ peptides are secreted from the endolysosome system, we treated HEK293 cells stably transfected with human APP, with tetanus toxin, a well-established inhibitor of exocytosis. Inhibition of exocytosis increased the Aβ levels in the cell body (Supplementary Fig. 7e), indicating that Aβ may be exocytosed, fitting with previous observations^34^. Hence, our data support that AEP interacts with APP and proteolytically processes it in the endolysosomal organelles.

### APP N-terminal ectodomain shed by AEP cleavage is neurotoxic

**Figure 5:**
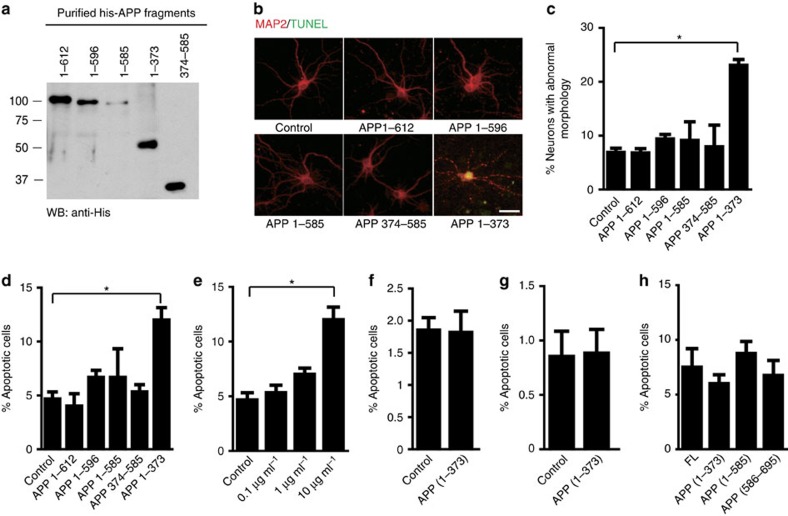
The AEP-fragmented APP_1–373_ induces neurodegeneration in primary cultured neurons. (**a**) Western blotting (WB) of the purified recombinant APP fragments. (**b**) Representative image of MAP2 and TUNEL staining after the neurons were incubated with APP fragments. Scale bar, 20 μm. (**c**) Percentage of neurons with abnormal morphology. Percentage of TUNEL-positive neurons induced by incubating with 10 μg ml^−1^ recombinant APP fragments (**d**) or different dose of APP_1–373_ fragment (**e**). Percentage of TUNEL-positive cells after PC12 cells (**f**) or HEK293 cells (**g**) were incubated with 10 μg ml^−1^ APP_1–373_ fragment (mean±s.e.m.). (**h**) Percentage of TUNEL-positive neurons after infected with lentivirus encoding APP fragments. Data are mean±s.e.m. of three independent experiments, and were analysed using *t*-test (**f**,**g**) or one-way analysis of variance followed by *post hoc* comparison (**c**,**d**,**e**,**h**), *P<0.01.

Accumulating evidence indicates that the ectodomain of APP cleavage may be tied to neurodegeneration in AD^35^,^36^. Next, we assessed whether the AEP-derived APP fragments influence neuronal viability. Strikingly, treatment of primary neurons with purified recombinant His-tagged APP_1–373_, mimicking the AEP-derived secreted APP fragment, triggered extensive axonal fragmentation and neuronal cell death. In contrast, sAPPα, sAPPβ, APP_1–585_ and APP_374–585_ fragments did not exhibit any demonstrable neurotoxic effect (Fig. 5a–c). TUNEL staining showed that the N-terminal APP_1–373_ fragment (10 μg ml^−1^) induced apoptosis (Fig. 5d,e). The APP_1–373_ fragment was not toxic to either PC12 cells or HEK293 cells (Fig. 5f,g), suggesting the toxic effect is specific to certain cell types, especially neurons. However, the AEP-derived APP fragments did not induce neuronal apoptosis when expressed within neurons (Fig. 5h), suggesting that only secreted N-terminal APP_1–373_ fragment is neurotoxic, presumably by binding to cell surface receptors. As shown earlier, the AEP cleavage of APP at N585 accelerates BACE1 processing of the resultant APP_586–695_ fragment. Hence, the combined effects of the two AEP cleavages of APP, leading to increased levels of the neurotoxic secreted APP_1–373_ and Aβ products, respectively, would be deleterious in AD.

### AEP is upregulated and activated during ageing and in AD

**Figure 6:**
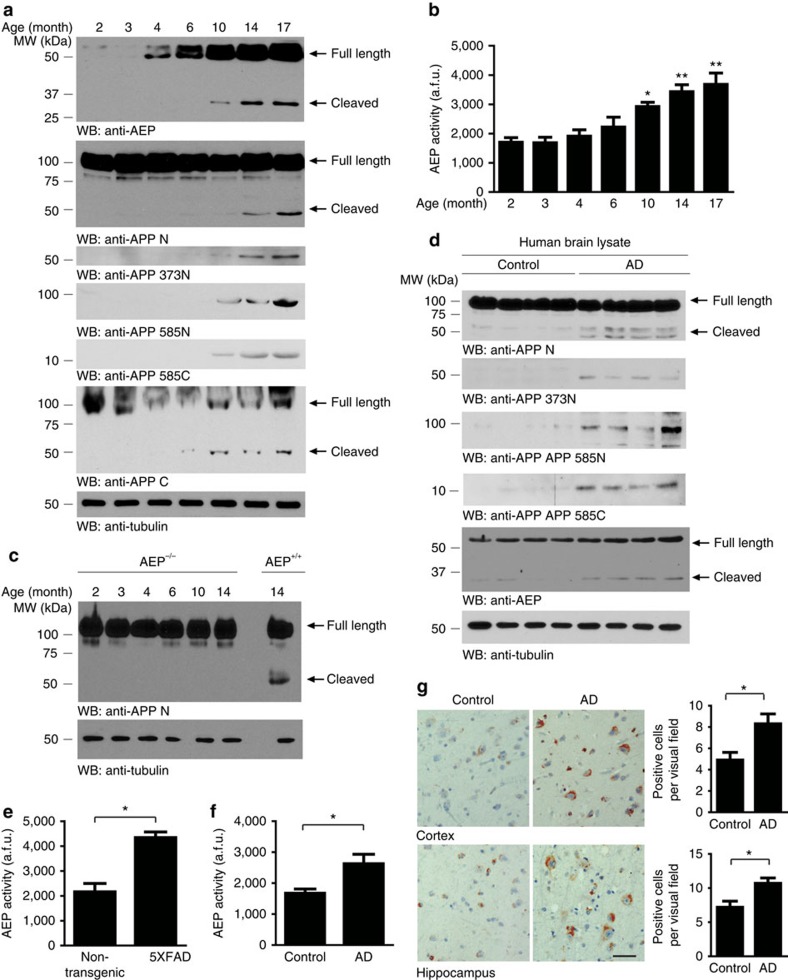
AEP is upregulated and cleaves APP during ageing and in AD. (**a**) Western blot (WB) showing AEP and APP expressing and processing in mouse brain during ageing. (**b**) AEP enzymatic activity analysis (mean±s.e.m.; *n*=6; one-way analysis of variance (ANOVA), *P<0.01 compared with 2-, 3- and 4-month-old mouse brain; **P<0.01 compared with 2-, 3-, 4- and 6-month-old mouse brain). (**c**) WB showing APP fragments in AEP^−/−^ mice brain. (**d**) WB detection of APP processing in human brain samples from AD patients and age-matched controls. (**e**) AEP activity in 6-month-old 5XFAD mice and non-transgenic controls (mean±s.e.m.; *n*=6; *t*-test, *P<0.01). (**f**) AEP activity in human brain samples from AD patients and age-matched controls (mean±s.e.m.; *n*=6; *t*-test, *P<0.01). (**g**) Immunostaining showing the presence of AEP-derived APP fragments in AD brain (mean±s.e.m.; *t*-test, *n*=8; *P<0.01). Scale bar, 50 μm. Data were analysed using one-way ANOVA followed by *post hoc* comparison.

The greatest known risk factor for AD is increasing age. We assessed AEP activity in ageing brain. AEP protein levels were barely detectable in the brain at 2 or 3 months of age, but escalated at 4 months and continued to increase substantially with age. AEP enzymatic activity is dependent on an autocleavage, with the activated component first detectable in mouse brain at 10 months of age, with further increases during ageing. There was a concomitant increase in the APP N-terminal and C-terminal fragments, with the AEP-derived APP_1–373_, APP_1–585_ and APP_586–695_ highly enriched and tightly coupled to the temporal pattern of AEP activation (Fig. 6a). AEP enzymatic activity was also increased in an age-dependent manner, correlating with the presence of the mature active AEP protein (Fig. 6b). We observed similar age-dependent increases in AEP activity and APP cleavage patterns in spinal cord (Supplementary Fig. 8a). The AEP-derived 50-kDa N-terminal immunoreactive APP fragment was completely abolished in AEP^−/−^ mice, even in the aged brain, supporting the hypothesis that APP processing during ageing is mediated by AEP (Fig. 6c). We further confirmed the cleavage of endogenous APP by knocking down AEP in HEK293 cells. The AEP-dependent cleavage fragments were found in control HEK293 cells, and were not detected when AEP was depleted by the siRNA (Supplementary Fig. 8b). In human brains, AEP-derived APP fragments APP_1–373_, APP_1–585_ and APP_586–695_ were all increased in AD cases compared with age-matched controls. Active AEP fragments were also increased in AD brains than control brains (Fig. 6d). Enzymatic analysis demonstrated that AEP activity was also substantially increased in brains from both the 5XFAD mouse model of AD and in human AD cases (Fig. 6e,f). Immunohistochemistry showed a marked increase in intraneuronal AEP-derived APP_586–695_ immunoreactivity in the cortex and hippocampus in AD cases (Fig. 6g). Although RNA-seq data suggest that AEP/LGMN is highly expressed in microglia^29^, immunohistochemistry staining shows that AEP immunoreactivity is also clearly enriched in neurons (Supplementary Fig. 8c). AEP and APP_586–695_ are also highly expressed in neurons of 5XFAD mouse brain as confirmed by confocal microscopy (Supplementary Fig. 8d,e). To investigate the relationship between the concentrations of AEP-derived APP fragments and Aβ, we developed an enzyme-linked immunosorbent assay (ELISA) using our cleavage-stie-specific antibodies and found that the concentrations of AEP-derived APP_586–695_ correlated with Aβ in AD and age-matched control brains (Supplementary Fig. 8f). Collectively, these findings indicate that AEP protein expression, autocleavage and enzyme activity are all upregulated during ageing, contributing to this novel APP processing event and increased Aβ production.

### Knockout of AEP reverses synaptic dysfunction in 5XFAD mice

**Figure 7:**
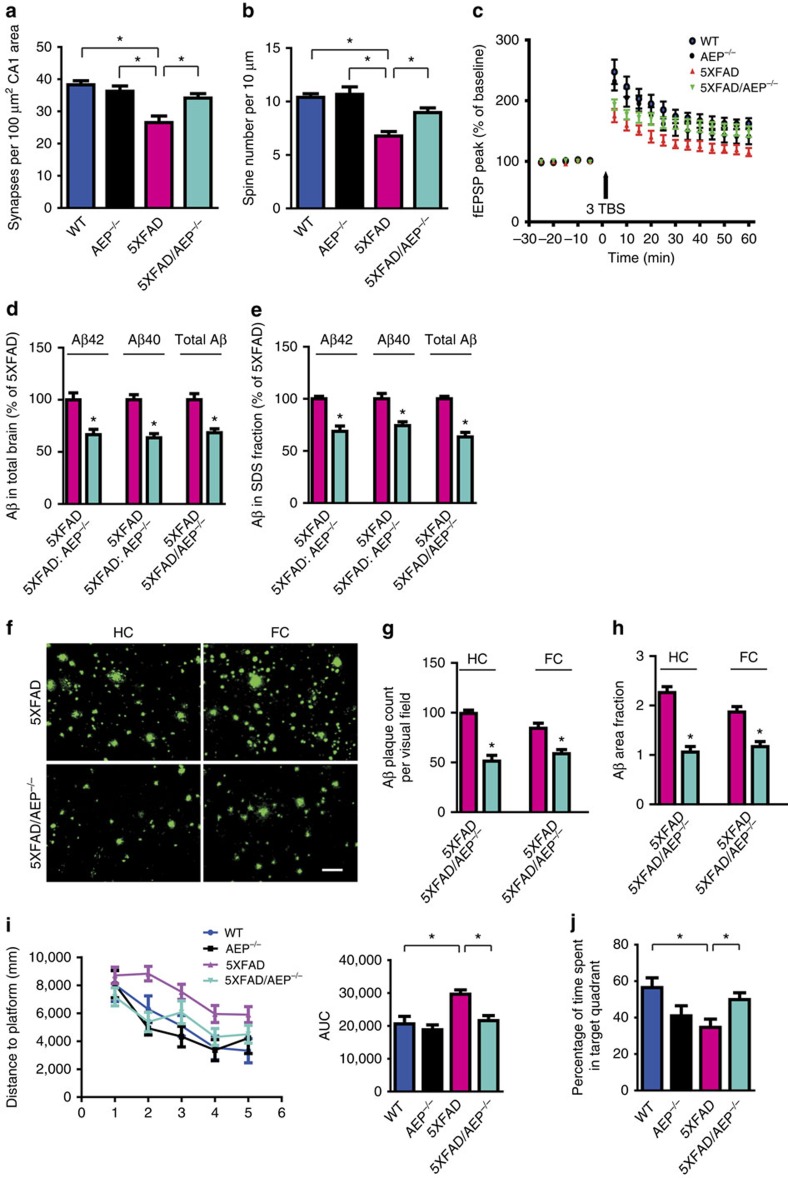
AEP gene deficiency ameliorates synaptic dysfunction, Aβ deposition and cognitive deficits in the 5XFAD mouse model. (**a**) The density of synapse determined by electron microscopy (mean±s.e.m.; *n*=6; *P<0.01). (**b**) Spine density in the hippocampus determined by Golgi staining (mean±s.e.m.; *n*=4; *P<0.01). (**c**) AEP deletion alleviates electrophysiological dysfunction in 5XFAD mice (mean±s.e.m.; *n*=6 in each group; *P<0.05). (**d**,**e**) ELISA quantification of Aβ in total brain lysates (**d**) or SDS fraction (**e**) from 6-month-old mice (mean±s.e.m.; *n*=9 in 5XFAD, *n*=10 in 5XFAD/AEP^−/−^ mice; *P<0.01). (**f**) Thioflavin-S staining showing the Aβ plaques in the hippocampus (HC) and frontal cortex (FC). Scale bar, 50 μm. (**g**,**h**) Quantification of number and surface area of Aβ plaques (mean±s.e.m.; *n*=6; *P<0.01). (**i**) Morris water maze analysis as distance travelled (millimetres) and integrated distance (area under the curve, AUC) for WT (*n*=8), AEP^−/−^ (*n*=8), 5XFAD (*n*=9) and 5XFAD/AEP^−/−^ (*n*=10) mice (mean±s.e.m.; *P<0.01). (**j**) Probe trial result. Shown is the mean±s.e.m. percentage of time spent in the target quadrant (*P<0.05). Data were analysed using *t*-test (**d**,**e**,**g**,**h**) or one-way analysis of variance followed by *post hoc* comparison (**a**,**b**,**c**,**i**,**j**). TBS, theta-burst stimulation.

Synaptic loss is believed to be the basis of cognitive impairment in the early phase of AD^37^. To assess the physiological role of AEP in this process, we crossed AEP^−/−^ mice with 5XFAD mice to generate 5XFAD/AEP^−/−^ mice (Supplementary Fig. 9a). In 5XFAD mice, significant synaptic loss and behaviour deficits were detected at 5 months of age, in the absence of detectable neuronal loss^38^. Electron microscopic analysis revealed significant synapse loss in the CA1 region in 5XFAD mice compared with the age-matched non-transgenic control mice and AEP^−/−^ mice. Deletion of AEP in 5XFAD mice (5XFAD/AEP^−/−^) rescued the synapse loss (Fig. 7a and Supplementary Fig. 10a). We also assessed the density of dendritic spines along individual Golgi-stained pyramidal neurons. Spine density was decreased in 5XFAD mice model, and this defect was reversed in 5XFAD/AEP^−/−^ mice (Fig. 7b and Supplementary Fig. 10b). 5XFAD mice display significantly impaired long-term potentiation (LTP) at Schaffer collateral-CA1 pathways^39^. Electrophysiological analysis demonstrated significantly increased LTP magnitude in 5XFAD/AEP^−/−^ mice when compared with 5XFAD mice. In contrast, LTP was comparable between the age-matched WT and AEP^−/−^ mice (Fig. 7c). Hence, these results show that inactivation of AEP rescues synaptic loss and LTP deficits in 5XFAD mice.

### Deletion of AEP ameliorates memory deficits in 5XFAD mice

To ascertain whether AEP influences the processing of APP and accumulation of Aβ peptides *in vivo*, we analysed total Aβ, Aβ40 and Aβ42 in the brains of 5XFAD/AEP^−/−^ mice. We found ∼30% reduction in all Aβ peptide species in 5XFAD/AEP^−/−^ mice compared with 5XFAD mice at 6 months of age. In addition, the highly aggregated, SDS-extractable forms of Aβ peptides were also decreased in 5XFAD/AEP^−/−^ mice (Fig. 7d,e). Remarkably, the cerebral Aβ level was lower in 5XFAD/AEP^−/−^ mice brain than in 5XFAD mice brain even at 1.5 months of age, before plaque deposition (Supplementary Fig. 10c). Thioflavin-S staining and Aβ immunohistochemistry both revealed fewer Aβ plaques in hippocampus and frontal cortex in 5XFAD/AEP^−/−^ versus 5XFAD mice. Quantification of the Aβ plaque number and surface area demonstrated substantially reduced plaque burden in 5XFAD/AEP^−/−^ mouse brain (Fig. 7f,g and Supplementary Fig. 10d,e). Immunoblotting analysis revealed that the AEP-derived APP fragments in 5XFAD mice were diminished in 5XFAD/AEP^−/−^ mice brain. APP and BACE1 expression levels were similar between 5XFAD and 5XFAD/AEP^−/−^ mice, but the C-terminal APP fragment (C99) resulting from BACE1 cleavage was decreased in 5XFAD/AEP^−/−^ mice. These results indicate that AEP deletion reduces Aβ production by attenuating BACE1-mediated cleavage of APP (Supplementary Fig. 9b).

Next, we evaluated spatial memory abilities in 6-month-old WT, AEP^−/−^, 5XFAD and 5XFAD/AEP^−/−^ mice using the Morris water maze test. As expected, 5XFAD mice showed longer latency periods and longer swim path distance than WT mice, representing deficits in spatial memory formation. However, 5XFAD/AEP^−/−^ mice were largely protected from spatial memory impairment (Fig. 7i and Supplementary Fig. 10f). All the mice exhibited comparable swim speeds, indicating that AEP gene knockout does not affect motor function (Supplementary Fig. 10g). 5XFAD/AEP^−/−^ mice also performed better than 5XFAD mice on the probe test, spending more time in the target quadrant and indicating better memory recall (Fig. 7j). To verify that the behavioural effects of 5XFAD/AEP^−/−^ were not strain dependent, we also examined the rescue effect of AEP gene knockout in another well-established AD mouse model expressing two AD mutant genes, *APP* and *PS1*. APP/PS1/AEP^−/−^ mice at 15 months of age showed shorter latency periods and swim path distance than APP/PS1 mice during the training sessions, and spent more time in the target quadrant during the probe test (Supplementary Fig. 11a–d). Thus, AEP gene knockouts protect against memory deficits in two different AD mouse models.

### Preserved memory in mice expressing uncleavable APP

**Figure 8:**
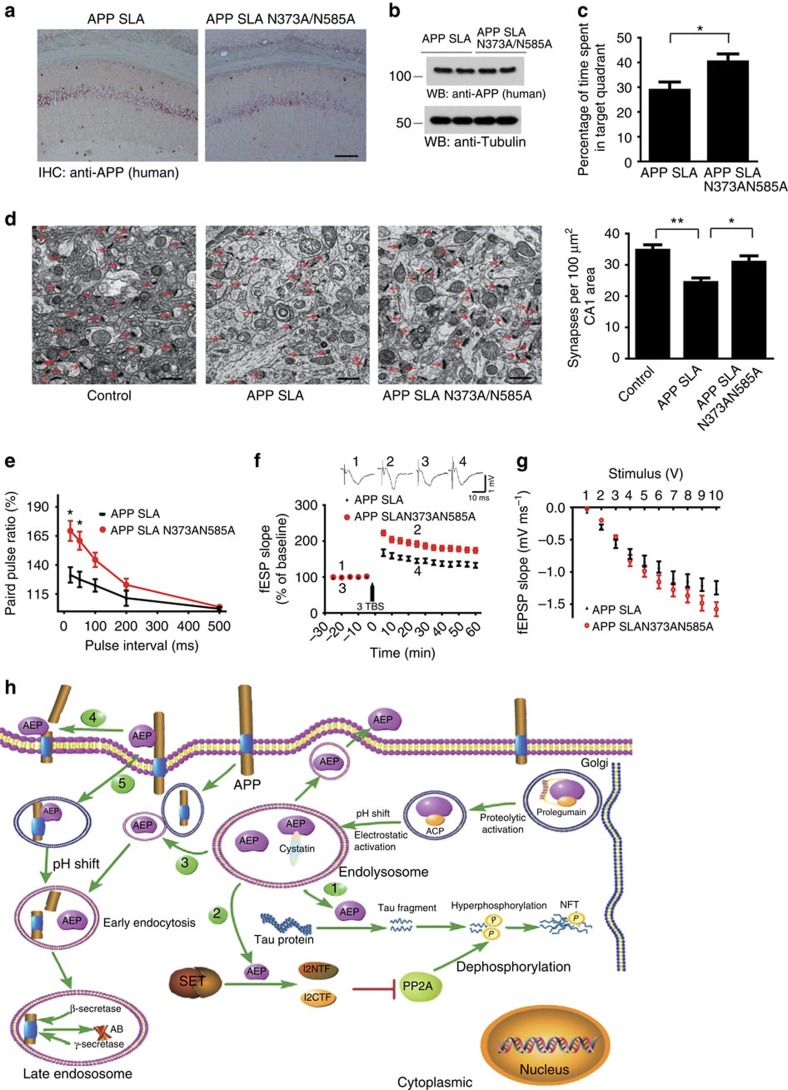
Blockade of AEP cleavage prevents synaptic and cognitive dysfunction induced by APP SLA. (**a**,**b**) AAV-mediated expression of human APP SLA and APP SLA N373AN585A detected by immunohistochemistry and western blot (WB) using an antibody specific to human APP. Scale bar, 100 μm. (**c**) Probe trial of Morris water maze test showing blockade of AEP cleavage prevents memory deficits induced by APP SLA (mean±s.e.m.; *P<0.05). (**d**) The synaptic density in the hippocampus of mice injected with AAV-APP SLA and AAV-APP SLA N373AN585A determined by electron microscopy (mean±s.e.m.; *n*=6; *P<0.05, **P<0.01). Scale bar, 1 μm. (**e**–**j**) Blockade of AEP cleavage preserves the electrophysiological function. The APP SLA N373AN585A mice showed higher ratio of paired pulse (**e**), higher averaged magnitude of LTP (**f**) and higher slope of I/O curve (**g**) than the APP SLA N373AN585A mice. (**h**) Possible pathways for AEP-mediated cleavage of APP, tau and SET in AD. (1) AEP might translocate from the endolysosome into the cytoplasmic space, where it cleaves tau; (2) intracellular AEP cuts SET, leading to PP2A inhibition and consequent tau hyperphosphorylation; (3) intracellular AEP can be fused with the endocytosed APP; (4) activated AEP might be secreted extracellularly, where it interacts with the ectodomain of APP; (5) AEP and APP can also form a complex, which can be internalized via endocytosis. Data were analysed using *t*-test (**c**,**e**,**f**,**g**) or one-way analysis of variance followed by *post hoc* comparison (**d**).

To confirm the role of AEP-mediated APP cleavage in the pathogenesis of AD, we injected WT mice with adeno-associated virus (AAVs) encoding human mutant APP_SLA_ expressing the Swedish, London and Austrian mutations that are associated with early-onset familial AD^40^or an AEP-uncleavable form APP_SLA/N373A/N585A_. The AAVs were injected into the hippocampus, where they expressed the embedded cDNA specifically in pyramidal neurons under control of the human synapsin-1 promoter. The expression levels of APP_SLA_ and APP_SLA/N373A/N585A_ were similar 6 months after the intracerebral injection of the AAVs as indicted by immunohistochemistry and western blot using an antibody specific to human APP (Fig. 8a,b). In the Morris water maze probe test, the APP_SLA/N373A/N585A_ mice spent more time in the target quadrant than did the APP_SLA_ mice, indicating preserved cognitive function (Fig. 8c). Expression of APP_SLA_ caused significant synaptic loss in the hippocampus, while the AEP-uncleavable N373A/N585A mutations ameliorated the synaptic loss (Fig. 8d). Electrophysiological analysis found that the paired pulse ratio was preserved in APP_SLA/N373A/N585A_ mice as compared with the APP_SLA_ mice (Fig. 8e). The magnitude of LTP was also significantly elevated in mice expressing uncleavable APP_SLA/N373A/N585A_ (Fig. 8f). Furthermore, the slope of I/O curve was preserved in the APP_SLA/N373A/N585A_ mice (Fig. 8g). Collectively, these results strongly support that the AEP-mediated processing of APP plays critical roles in the pathogenesis of AD.

## Discussion

In the present study, we have identified AEP as a novel pro-amyloidogenic protease cleaving the APP ectodomain at both N373 and N585 sites. AEP cleavage of APP at N585 enhances subsequent BACE1 processing of the AEP-generated APP stub (APP_586–695_), increasing Aβ levels. The AEP-generated N-terminal ectodomain of APP_1–373_ triggers neurodegeneration, indicating that AEP activation may induce neuronal cell death through proteolytic processing of APP. Blockade of AEP processing of APP in a variety of paradigms protects against Aβ accumulation, synaptic loss and behavioural deficits. Since AEP expression and activation is elevated in brain in an age-dependent manner, and leads to increased cleavage of APP in aged brains (Fig. 6), we propose that AEP contributes to the strong effect of ageing on AD risk. Collectively, these observations provide a rationale for the development of AEP inhibitors for treating AD.

The accumulation of Aβ in AD brain is caused by an imbalance between its production and clearance^41^. There is direct evidence that Aβ clearance is impaired in late-onset sporadic AD^42^. On the other hand, there is also evidence demonstrating increased production of Aβ in familial Alzheimer's disease caused by Swedish mutation of APP^43^. Late-onset sporadic AD cases show elevated BACE1 levels, which is the rate-limiting enzyme for Aβ production. The elevation of BACE1 activity is correlated with brain Aβ loads^44^,^45^. Since human clinical trials of improving Aβ clearance for AD have failed^46^,^47^, Aβ clearance defects might not be the major or sole contributor to AD onset and progression. Our study supports a pathogenic model in which AEP levels and activity increase with age, creating a novel cleavage of APP that increases BACE1 processing and Aβ production under physiological conditions in neurons *in vitro* and *in vivo*.

APP is processed by α-, β- and γ-secretases, generating amyloidogenic and non-amyloidogenic fragments. Secretase-independent processing pathways may also exist, in that the half-life of APP is very short and since not all APP is secreted^48^. APP is also a substrate of caspases^30^,^49^, but the impact of this processing on Aβ generation and/or AD pathology is still not well established^50^. APP is degraded in the lysosomes^51^,^52^, presumably mediated at least in part by lysosomal enzymes such as AEP or cathepsins. Interestingly, cathepsin B cleaves APP at β-secretase cleavage site^31^,^53^,^54^. Here we provide both molecular and biochemical evidence showing that AEP may act as a novel δ-secretase that mediates the proteolytic processing of APP.

Where does AEP processing APP occur? It has been shown previously that lysosomal enzymes are abnormally distributed in human AD patient brains^55^. AEP usually resides in endolysosomes in healthy cells, but it is secreted extracellularly or may be leaked into the cytoplasm in cancer cells, aged cells and in AD neurons^21^,^26^. We found that AEP co-fractionates with BACE1 and APP along with endolysosomal markers and that it binds APP in mouse brain (Fig. 4). Fitting with these observations, Aβ treatment also elicits AEP co-localization with APP in primary neurons (Fig. 4c). AEP is secreted extracellularly, and associates with APP on the cell surface. Cell surface-associated AEP can be internalized and processed to its active form (Supplementary Fig. 7).

Given that APP cleavage by AEP promotes the subsequent BACE1 processing of APP_586–695_ via release of steric hindrance and more access to the substrate, AEP might act as an upstream/initiating trigger for APP amyloidogenic processing. Since Aβ processing is mainly mediated by BACE1 and γ-secretase, deletion of the upstream δ-secretase predominantly impairs APP cleavage efficiency by BACE1. In addition to APP, AEP also cleaves other substrates implicated in AD including tau and SET (I_2_^PP2A^)^19^,^21^,^23^. The reduction of Aβ levels in 5XFAD/AEP^−/−^ mice rescues spine density, LTP and cognition (Fig. 7).

On the basis of these findings, we propose the following model. During ageing and other cellular stressors in the AD brain, AEP translocates from the endolysosome into the cytoplasmic space, where it cleaves tau, resulting in truncated neurotoxic fragments, hyperphosphorylation and neurofibrillar tangle formation^23^. Moreover, AEP cleaves SET, leading to PP2A inhibition and accelerates tau hyperphosphorylation^21^. AEP is also secreted extracellularly and distributes in the CSF (Supplementary Fig. 7a). During the ageing process and in AD, the reduction in brain pH decreases and reduced cystatin C levels both promote AEP protease enzymatic activation. The secreted AEP binds APP on the plasma membrane and cleaves APP at N373 and N585 residues, shedding a neurotoxic APP_1–373_ fragment. The AEP/APP complex may also be internalized via endocytosis, with the AEP further activated in the low-pH environment of the endosome, cleaving APP at the N585 site. The resultant APP_586–695_ fragment subsequently serves as an optimal substrate for BACE1, augmenting amyloid formation (Fig. 8h). Consequently, the activated AEP and reduced inhibitory cystatin C levels in AD brains coordinately result in amyloid deposition and aggregation.

AEP expression and activity have been linked to a number of pathological conditions including atherosclerosis, cancer, stroke and neurodegenerative diseases^19^,^21^,^56^,^57^,^58^. Despite large-scale efforts to therapeutically target putative disease mechanisms in AD, such as Aβ production or clearance, neuroprotective treatments are still lacking. There is growing consensus that gaining a better understanding of the underlying disease mechanisms is urgently needed. Our findings provide substantial *in vitro* and *in vivo* evidence demonstrating that blockade of AEP cleavage of APP reduces Aβ production and amyloid deposition, underscoring that δ-secretase activity by AEP plays a crucial role in APP metabolism and in AD pathogenesis. Since δ-secretase cleaves both APP and tau in an age-dependent manner and mediates the amyloid plaque and NFT pathology onset^23^, our findings highlight a previously unappreciated role of this novel secretase in AD progression. Because this enzyme also cleaves TDP-43 (ref. 22), this δ-secretase might also contribute to other age-dependent neurodegenerative diseases including some forms of frontotemporal lobar degeneration and amyotrophic lateral sclerosis. Hence, inhibition of AEP may be a novel therapeutic strategy for treating several pathogenic mechanisms that contribute to AD and other neurodegenerative diseases.

## Methods

### Transgenic mice

5XFAD mice and APP/PS1 mice on a C57BL/6J background were obtained from the Jackson Laboratory (Stock No. 006554 and 004462, respectively). AEP knockout mice on a mixed C57BL/6 and 129/Ola background were generated as reported^18^. Only male mice were used. All mice were housed under standard conditions at 22 °C and a 12-h light:dark cycle with free access to food and water. Animal care and handling was performed according to the Declaration of Helsinki and the Emory Medical School Guidelines. The following animal groups were analysed: WT, AEP^−/−^, 5XFAD, 5XFAD/AEP^−/−^, APP/PS1 and APP/PS1/AEP^−/−^. Sample size was determined by Power and Precision (Biostat). Investigators were blinded to the group allocation during the animal experiments. The protocol was reviewed and approved by the Emory Institutional Animal Care and Use Committee.

### Human tissue samples

Post-mortem brain samples were dissected from frozen brains of eight AD cases (age 74.5±11.2 years, mean±s.d.) and eight non-demented controls (age 73.9±12.7 years) from the Emory Alzheimer's Disease Research Center. Informed consent was obtained from all subjects. The study was approved by the Emory University CND Tissue Committee. AD was diagnosed according to the criteria of the Consortium to Establish a Registry for AD and the National Institute on Aging. Diagnoses were confirmed by the presence of amyloid plaques and neurofibrillary tangles in formalin-fixed tissue. The post-mortem interval was similar between the AD group and control group.

### Antibodies and reagents

Antibodies to the following targets were used: anti-APP N-terminal antibody (clone 22C11, 1:1,000, Calbiochem), myc (1:1,000, Calbiochem), anti-APP C-terminal antibody (1:500, CT15, from Dr Edward Koo, University of California^59^), GFP (1:1,000, Santa Cruz), and LAMP1 (1:500, Santa Cruz), GST-horseradish peroxidase (HRP), β-tubulin III, tubulin, MAP2 and Aβ (all 1:1,000, Sigma-Aldrich), presenilin-1 (1:500, Abcam), BACE1, (1:1,000, Cell Signaling), GGA3 (1:500, Cell Signaling), EEA1 (1:500, Cell Signaling), AEP antibody clone 6E3 (1:1,000, from Dr Colin Watts, University of Dundee^17^), human AEP antibody (1:200, R&D) and sAPPα (1:1,000, MBL). Inhibitors were used against: caspase (20 μM ZVAD-fmk, Calbiochem), α-secretase (10 μM TAPI-1, Calbiochem), β-secretase (50 nM KTEETSEVN(stat)VAEF, Calbiochem), γ-secretase (25 μM DAPT, Calbiochem) and cathepsin (10 μM E64, Sigma-Aldrich). Histostain-SP, mouse and human Aβ40 and Aβ42 ELISA kits were purchased from Invitrogen. Total Aβ chemiluminescent ELISA kit was purchased from Covance. The *In Situ* Cell Death Detection Kit was purchased from Roche. Recombinant AEP was purchased from Novoprotein. The recombinant AEP was first activated by incubation in activation buffer (0.1 M NaOAc, 0.1 M NaCl, pH 4.5) at 37 °C for 4 h. Recombinant BACE1 was purchased from Sigma-Aldrich. Recombinant ADAM10 was purchased from R&D. Z-Ala-Ala-Asn-AMC was purchased from Bachem. All chemicals not included above were purchased from Sigma-Aldrich.

### *In vitro* APP cleavage assay

To assess the cleavage of APP by AEP *in vitro*, HEK293 cells (obtained from the American Type Culture Collection (ATCC)) were transfected with 10 μg GFP-APP or GST-APP plasmids by the calcium phosphate precipitation method. Forty-eight hours after transfection, the cells were collected, washed once in PBS, lysed in lysis buffer (50 mM sodium citrate, 5 mM dithiothreitol (DTT), 0.1% CHAPS and 0.5% Triton X-100, pH 7.4), and centrifuged for 10 min at 14,000*g* at 4 °C. The supernatant was then incubated with mouse kidney lysates at pH 7.4 or 6.0 at 37 °C for 30 min. To measure the cleavage of purified APP fragments by AEP, BACE1 or ADMA10, GST-tagged APP full length or fragments were purified with glutathione beads. The purified APP was incubated with recombinant AEP (5 μg ml^−1^) in AEP buffer (50 mM sodium citrate, 5 mM DTT, 0.1% CHAPS and 0.5% Triton X-100, pH 6.0), recombinant BACE1 (100 U ml^−1^) in BACE1 buffer (20 mM sodium acetate, 0.1% Triton X-100, pH 4.5), or recombinant ADAM10 (1 ng μl^−1^) in ADAM10 buffer (25 mM Tris, 2 μM ZnCl_2_, 0.005% Brij-35, pH 9.0) for 30 min. The samples were then boiled in 1 × SDS loading buffer and analysed by immunoblotting.

### AEP activity assay

Tissue homogenates or cell lysates (10 μg) were incubated in 200 μl assay buffer (20 mM citric acid, 60 mM Na_2_HPO_4_, 1 mM EDTA, 0.1% CHAPS and 1 mM DTT, pH 6.0) containing 20 μM AEP substrate Z-Ala-Ala-Asn-AMC (Bachem). AMC released by substrate cleavage was quantified by measuring at 460 nm in a fluorescence plate reader at 37 °C for 1 h in kinetic mode. The activity of AEP was expressed as the reading at 1 h minus the first reading.

### Western blot analysis

The mouse brain tissue or human tissue samples were lysed in lysis buffer (50 mM Tris, pH 7.4, 40 mM NaCl, 1 mM EDTA, 0.5% Triton X-100, 1.5 mM Na_3_VO_4_, 50 mM NaF, 10 mM sodium pyrophosphate and 10 mM sodium β-glycerophosphate, supplemented with protease inhibitors cocktail), and centrifuged for 15 min at 16,000*g*. The supernatant was boiled in SDS loading buffer. After SDS–PAGE, the samples were transferred to a nitrocellulose membrane. Western blot analysis was performed with a variety of antibodies. Images have been cropped for presentation. Full-size images are presented in Supplementary Fig. 12.

### Co-immunoprecipitation

The mouse brain tissue samples were lysed in lysis buffer and centrifuged for 15 min at 16,000*g*. The supernatant was incubated with anti-AEP antibody and protein A/G-agarose overnight at 4 °C. After extensive washing, the bound proteins were eluted from the beads by boiling in Laemmli sample buffer and subjected to western blot analyses.

### Subcellular fractionation

Subcellular fractionation of mouse brain tissues was performed as described previously^60^. Briefly, 150 mg frontal cortex tissue was minced with a scalpel blade in 1 ml of homogenization buffer (0.25 M sucrose, 1 mM MgCl_2_, 10 mM Tris-HCl, pH 7.4, supplemented with protease inhibitor cocktail). The buffer was discarded after centrifugation at 100*g* for 2 min. The tissues were suspended in 1.5 ml homogenization buffer and homogenized by successive passages through needles of increasing gauge number (19–26). The suspension was centrifuged at 1,000*g* for 10 min to discard nuclei and cellular debris. The supernatant was adjusted to 1.4 M sucrose, and incorporated into a discontinuous sucrose gradient consisting of the four following layers: 2 ml 2 M sucrose, 2.25 ml 1.4 M sucrose, 3.75 ml 1.2 M sucrose and 5 ml 0.8 M sucrose. The gradients were centrifuged for 2.5 h at 100,000* g*. Thirteen fractions were collected from the top of the gradient and stored as aliquots at −80 °C. Equal volumes of each fraction were boiled in 1 × SDS loading buffer and analysed by immunoblotting.

### Mass spectrometry analysis

Protein samples were in-gel digested with trypsin. Peptide samples were resuspended in loading buffer (0.1% formic acid, 0.03% trifluoroacetic acid and 1% acetonitrile) and loaded onto a 20-cm nano-high-performance liquid chromatography column (internal diameter 100 μm) packed with Reprosil-Pur 120 C18-AQ 1.9 μm beads (Dr. Maisch) and eluted over a 2 h 4–80% buffer B reverse phase gradient (buffer A: 0.1% formic acid and 1% acetonitrile in water; buffer B: 0.1% formic acid in acetonitrile) generated by a NanoAcquity UPLC system (Waters Corporation). Peptides were ionized with 2.0 kV electrospray ionization voltage from a nano-ESI source (Thermo) on a hybrid LTQ XL Orbitrap mass spectrometer (Thermo). Data-dependent acquisition of centroid MS spectra at 30,000 resolution and MS/MS spectra were obtained in the LTQ following collision-induced dissociation (collision energy 35%, activation Q 0.25, activation time 30 ms) for the top 10 precursor ions with charge determined by the acquisition software to be *z*≥2. Dynamic exclusion of peaks already sequenced was for 20 s with early expiration for two count events with signal to noise >2. Automatic gating control was set to 150 ms maximum injection time or 106 counts. To identify AEP cleavage sites on human APP, the SageN Sorcerer SEQUEST 3.5 algorithm was used to search and match MS/MS spectra to a complete semi-tryptic human proteome database (NCBI reference sequence revision 50, with 66,652 entries) plus pseudo-reversed decoys sequences^61^,^62^ with a 20 p.p.m. mass accuracy threshold. Only *b-* and *y*-ions were considered for scoring (Xcorr) and Xcorr along with ΔCn were dynamically increased for groups of peptides organized by a combination of trypticity (fully or partial) and precursor ion charge state to remove false-positive hits along with decoys until achieving a false-discovery rate (FDR) of <5% (<0.25% for proteins identified by more than one peptide). The FDR was estimated by the number of decoy matches (nd) and total number of assigned matches (nt). FDR=2 × nd/nt, assuming mismatches in the original database were the same as in the decoy database. To detect the AEP-derived APP fragments, AD brain lysates were immunoprecipitated with anti-APP N-terminal antibody and subjected to proteomic analysis. All semi-tryptic MS/MS spectra for putative AEP-generated APP cleavage sites were manually inspected.

### Immunohistochemistry

Free-floating 30-μm-thick serial sections were treated with 0.3% hydrogen peroxide for 10 min. Then, sections were washed three times in PBS and blocked in 1% BSA and 0.3% Triton X-100, for 30 min followed by overnight incubation with primary antibodies at 4 °C. The signal was developed using Histostain-SP kit according to the manufacturer's instructions.

### Aβ plaque histology

Amyloid plaques were stained with Thioflavin-S. Free-floating 40-μm brain sections were incubated in 0.25% potassium permanganate solution for 20 min, rinsed in distilled water and incubated in bleaching solution containing 2% oxalic acid and 1% potassium metabisulfite for 2 min. After rinsed in distilled water, the sections were transferred to blocking solution containing 1% sodium hydroxide and 0.9% hydrogen peroxide for 20 min. The sections were incubated for 5 s in 0.25% acidic acid, then washed in distilled water and stained for 5 min with 0.0125% Thioflavin-S in 50% ethanol. The sections were washed with 50% ethanol and placed in distilled water. Then the sections were covered with a glass cover using mounting solution and examined under a fluorescence microscope. The plaque number and plaque area were calculated using ImageJ software (National Institutes of Health).

### Generation of AEP-derived APP fragment antibodies

The anti-APP 373N, anti-APP585N and anti-APP 585C antibodies were generated by immunizing rabbits with the following peptides: Ac-CESLEQEAAN-OH (anti-APP373N), Ac-CTRPGSGLTN-OH (anti-APP 585N) and H2N-IKTEEISEVC-amide (anti-APP 585C), respectively. The antiserum was pooled and the titres against the immunizing peptide were determined by ELISA. The maximal dilution giving a positive response using chromogenic substrate for HRP was >1:30,000. The immunoactivity of the antiserum was further confirmed by western blot and immunohistochemistry.

### Electron microscopy

Synaptic density was determined by electron microscopy. After deep anaesthesia, mice were perfused transcardially with 2% glutaraldehyde and 3% paraformaldehyde in PBS. Hippocampal slices were postfixed in cold 1% OsO_4_ for 1 h. Samples were prepared and examined using standard procedures. Ultrathin sections (90 nm) were stained with uranyl acetate and lead acetate, and viewed at 100 kV in a JEOL 200CX electron microscope. Synapses were identified by the presence of synaptic vesicles and postsynaptic densities.

### Golgi staining

Mouse brains were fixed in 10% formalin for 24 h, and then immersed in 3% potassium bichromate for 3 days in the dark. The solution was changed each day. Then the brains were transferred into 2% silver nitrate solution and incubated for 24 h in the dark. Vibratome sections were cut at 60 μm, air dried for 10 min, dehydrated through 95 and 100% ethanol, cleared in xylene and coverslipped.

### Electrophysiology

Acute hippocampal transversal slices were prepared from 6-month-old WT, AEP^−/−^, 5XFAD and 5XFAD/AEP^−/−^ mice as described previously^63^. Briefly, mice hippocampal slices were placed in a recording chamber (RC-22C, Warner Instruments) on the stage of an upright microscope (Olympus CX-31) and perfused at a rate of 3 ml min^−1^ with a-CSF (containing 1 mM MgCl_2_) at 23–24 °C. A 0.1 MΩ tungsten monopolar electrode was used to stimulate the Schaffer collaterals. The field excitatory postsynaptic potentials (fEPSPs) were recorded in CA1 stratum radiatum by a glass microelectrode filled with a-CSF with resistance of 3–4 MΩ. The stimulation output (Master-8; AMPI, Jerusalem) was controlled by the trigger function of an EPC9 amplifier (HEKA Elektronik, Lambrecht, Germany). fEPSPs were recorded under current-clamp mode. Data were filtered at 3 kHz and digitized at sampling rates of 20 kHz using Pulse software (HEKA Elektronik). The stimulus intensity (0.1 ms duration, 3–4.5 V) was set to evoke 40% of the maximum fEPSP and the test pulse was applied at a rate of 0.033 Hz. LTP of fEPSPs was induced by three theta-burst stimulation, it is four pulses at 100 Hz, repeated three times with a 200-ms interval).

### Mice brain tissue preparation and protein extraction

After completion of the behavioural test, mice were deeply anaesthetized with pentobarbital and transcardially perfused with saline, and the brains were rapidly removed. One hemisphere was fixed in 4% phosphate-buffered paraformaldehyde, while the other was snap frozen for biochemical analysis. For brain protein extraction, hemispheres were first extracted in RIPA buffer (25 mM Tris-HCl, pH 7.5, 150 mM NaCl, 1% NP-40, 0.5% NaDOC and 0.1% SDS), centrifuged at 100,000 r.p.m. for 30 min and the pellet containing insoluble Aβ was further extracted in 2% SDS, 25 mM Tris-HCl, pH 7.5.

### ELISA quantification of Aβ

To detect the concentration of Aβ in total brain lysates, the mouse brains were homogenized in 8 × mass of 5 M guanidine HCl/50 mM Tris-HCl (pH 8.0), and incubated at room temperature for 3 h. Then the samples were diluted with cold reaction buffer (PBS with 5% BSA and 0.03% Tween-20, supplemented with protease inhibitor cocktail), and centrifuged at 16,000*g* for 20 min at 4 °C. The Aβ in the total brain and the Aβ in the SDS fraction were analysed with human Aβ42 (KHB3441, Invitrogen), Aβ40 (KHB3481, Invitrogen) and total Aβ (SIG-38966, Covance) ELISA kits according to the manufacturer's instructions. The Aβ concentrations were determined by comparison with the standard curve. To assess the effect of AEP on Aβ production, HEK293 cells stably transfected with human APP695 were transfected with small interfering RNA (siRNA) against AEP using lipofection 2000 reagent. AEP 27mer siRNA was purchased from Origene. The siRNA sequences are rCrCrArUrGrGrArUrCrUrArCrUrGrGrArArUrArCrUrGrGTT, ArGrCrGrUrCrArArCrUrGrGrArUrGrGrArArGrArUrUrCGG. Non-targeting control siRNA (rCrGrUrUrArArUrCrGrCrGrUrArUrArArUrArCrGrCrGrUrArT) was transfected in parallel as control. To detect the Aβ production by different APP fragments, HEK293 cells were transfected with GST-APP full length, GST-APP_374–695_, GST-APP_586–695_, GFP-APP, GFP-APP N373A and GFP-APP N585A, respectively. Twenty-four hours post-transfection, cells were fed with fresh media. Media were collected after conditioning for 24 h, and cell debris was removed by centrifugation. Complete protease inhibitor cocktail (Roche) was added and Aβ40 and Aβ42 were quantified with human Aβ42 and Aβ40 ELISA kits (KHB3441 and KHB3481, Invitrogen). The concentrations of Aβ42 and Aβ40 in AEP^+/+^ and AEP^−/−^ mouse neuronal medium were analysed using mouse Aβ42 and Aβ40 ELISA kits (KMB3441 and KMB3481, Invitrogen).

### ELISA quantification of APP fragments

The ELISA was carried out by using 96-well Nunc-Immuno MaxSorp platesfor TMB (3,3′,5,5′-tetramethylbenzidine from Sigma Catalogue # T5525). These plates were coated with 100 μl antibody that specifically recognize AEP-derived APP fragment_586–695_ (1:200). The plates were incubated overnight at 4 °C and then washed once with 250 μl of PBS/Tween-20 (PBST; 0.5%, v/v) for 1 min and removed; the coated plates were blocked by adding 200 μl per well of 2% BSA in PBST for 2 h at room temperature. After blocking, 100 μl per well of samples diluted in 2% BSA/PBST (+2 mM Na_3_VO_4_) were added to each well and the plate was incubated overnight at 4 °C and washed four times with 250 ml of PBST next day. After washing, 100 μl per well of the CT15 that was diluted with ratio of 1:1,000 in 2% BSA/PBST was added and incubated overnight at 4 °C and then washed. After rinsing with PBST for three or four times, 100 μl per well of anti-mouse HRP-conjugated secondary antibody (Fisher) 1:5,000 diluted in 2% BSA/PBST was added and incubated for 2 h at room temperature and then rinsed with 250 ml PBST for three times. After the final wash, 100 ml of the substrate solution (TMB solution: to dissolve TMB 1 mg tablet in 1 ml dimethylsulfoxide, and freshly dilute to 9 ml pH 5.0 citrate buffer (to 50 ml de-ionized water, add 20.5 ml 0.1 M citric acid plus 29.5 ml 0.1 M sodium citrate, mix well) and add 100 ml 3% H_2_O_2_) was added to each well and incubated at 37 °C for 10–30 min. The reaction was stopped by adding 50 μl per well of 3 N HCl and the values of each well were recorded using microplate reader at 450 nm or luminescent, and subtract the readings at 650 nm from the readings at 450 nm. This subtraction will correct the optical imperfections in the plate.

### Morris water maze

Six-month-old WT, AEP^−/−^, 5XFAD, 5XFAD/AEP^−/−^ mice and 15-month-old APP/PS1 and APP/PS1/AEP^−/−^ mice were trained in a round, water-filled tub (52 inch diameter) in an environment rich with extra maze cues. An invisible escape platform was located in a fixed spatial location 1 cm below the water surface independent of a subject's start position on a particular trial. In this manner, subjects needed to utilize extra maze cues to determine the platform's location. At the beginning of each trial, the mouse was placed in the water maze with their paws touching the wall from 1 of 4 different starting positions (N, S, E and W). Each subject was given four trials per day for 5 consecutive days with a 15-min inter-trial interval. The maximum trial length was 60 s and if subjects did not reach the platform in the allotted time, they were manually guided to it. On reaching the invisible escape platform, subjects were left on it for an additional 5 s to allow for survey of the spatial cues in the environment to guide future navigation to the platform. After each trial, subjects were dried and kept in a dry plastic holding cage filled with paper towels to allow the subjects to dry off. The temperature of the water was monitored every hour so that mice were tested in water that was between 22 and 25 °C. Following the 5 days of task acquisition, a probe trial was presented during which time the platform was removed and the percentage of time spent in the quadrant, which previously contained the escape platform during task acquisition was measured over 60 s. All trials were analysed for latency and swim speed by means of MzeScan (Clever Sys, Inc.).

### Immunofluorescence

Cultured neurons were fixed in 4% formaldehyde for 10 min, washed with PBS, blocked in 1% BSA and 0.3% Triton X-100 for 30 min followed by overnight incubation with primary antibodies at 4 °C. The slides were washed three times in PBS and incubated with Texas Red-conjugated anti-mouse IgG and fluorescein isothiocyanate-conjugated anti-rabbit IgG for 1 h at room temperature. The slides were washed three times in PBS, then the sections were covered with a glass cover using mounting solution and examined using fluorescence microscopy or confocal microscopy (Olympus).

### Primary neuron culture

Primary neurons were dissected from E18 embryos and cultured as described previously^63^. To measure the effect of APP fragments on neurons, primary cortical neurons cultured 12 days *in vitro* (DIV 12) were exposed to different concentrations of APP fragments for 24 h, the neurons were then fixed in 4% formaldehyde, permeabilized and immunostained with anti-MAP2 antibody (1:1,000). Pictures of the neurons were taken by fluorescence microscopy. Neuronal apoptosis was detected with the *In Situ* Cell Death Detection Kit. The apoptotic index was expressed as the percentage of TUNEL-positive neurons out of the total number of MAP2-positive neurons. To detect the effect of APP_1–373_ on HEK293 cells and PC12 cells (from ATCC), the cells were incubated with APP_1–373_ at 10 μg ml^−1^ for 24 h. The cells were fixed and stained with *In* situ Cell Death Detection Kit and 4,6-diamidino-2-phenylindole. The percentage of TUNEL-positive nucleus out of the total nucleus was calculated.

### AAV vector packaging

pAAV vectors carrying human APP SLA were gifts from Dr Kuegler from Max Planck Institute of Psychiatry, Germany^40^. The vectors use the human synapsin I promoter to drive neuron-specific gene expression. All of the mutations were introduced using site-directed mutagenesis kit (Agilent Technologies). The AAV particles were prepared by Viral Vector Core at Emoy University.

### Stereotaxic injection of the virus

Three-month-old WT C57BL/6J mice were anaesthetized with phenobarbital (75 mg kg^−1^). Bilateral intracerebral injection of AAV-APP SLA and AAV-APP SLA N373AN585A was performed stereotactically at coordinates posterior 1.94 mm, lateral 1.4 mm and ventral 2.2 mm relative to bregma. A volume of 2 μl of viral suspension containing 2 × 10^11^ vector genome was injected in to each point using 10-μl glass syringes with a fixed needle at a rate of 0.5 μl min^−1^. The needle was remained in place for 5 min before it was removed slowly (throughout 2 min). The mice were placed on heating pad until it began to recover from the surgery.

### Statistical analysis

All the quantitative data were presented as mean±s.e.m. Statistical analysis was performed using either Student's *t*-test (two-group comparison) or one-way analysis of variance (more than two groups) followed by *post hoc* comparison, and differences with *P* values <0.05 were considered significant.

## Additional information

**How to cite this article:** Zhang, Z. *et al.* Delta-secretase cleaves amyloid precursor protein and regulates the pathogenesis in Alzheimer's disease. *Nat. Commun.* 6:8762 doi: 10.1038/ncomms9762 (2015).

## Supplementary Material

Supplementary Information Supplementary Figures 1-12
