# Supplementary material for: Delta-secretase cleaves amyloid precursor protein and regulates the pathogenesis in Alzheimer's disease
Source: Nat Commun. 2015 Nov 9;6:8762. doi: 10.1038/ncomms9762 (PMC4659940; doi:10.1038/ncomms9762)
Supplement: Supplementary Information — Supplementary Figures 1-12 [file ncomms9762-s1.pdf]

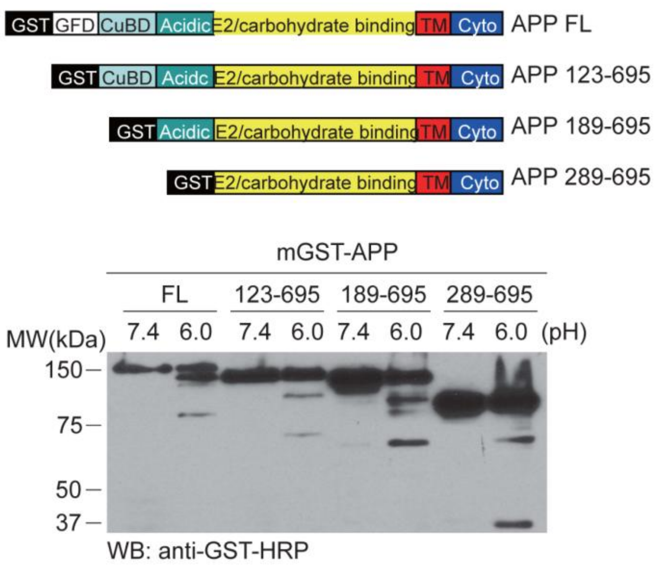

Supplementary Figure 1. APP cleavage assay. HEK293 cells were transfected with various GST-tagged N-terminal truncated APP fragments including GST-APP full-length (FL), APP (123-695), APP (189-695), or APP (289-695). The cell lysates were incubated with mouse kidney lysates at pH 7.4 or pH 6.0 at 37℃ for 30 min. APP cleavage was analyzed by Western blot. All of the APP fragments were cleaved at pH 6.0.

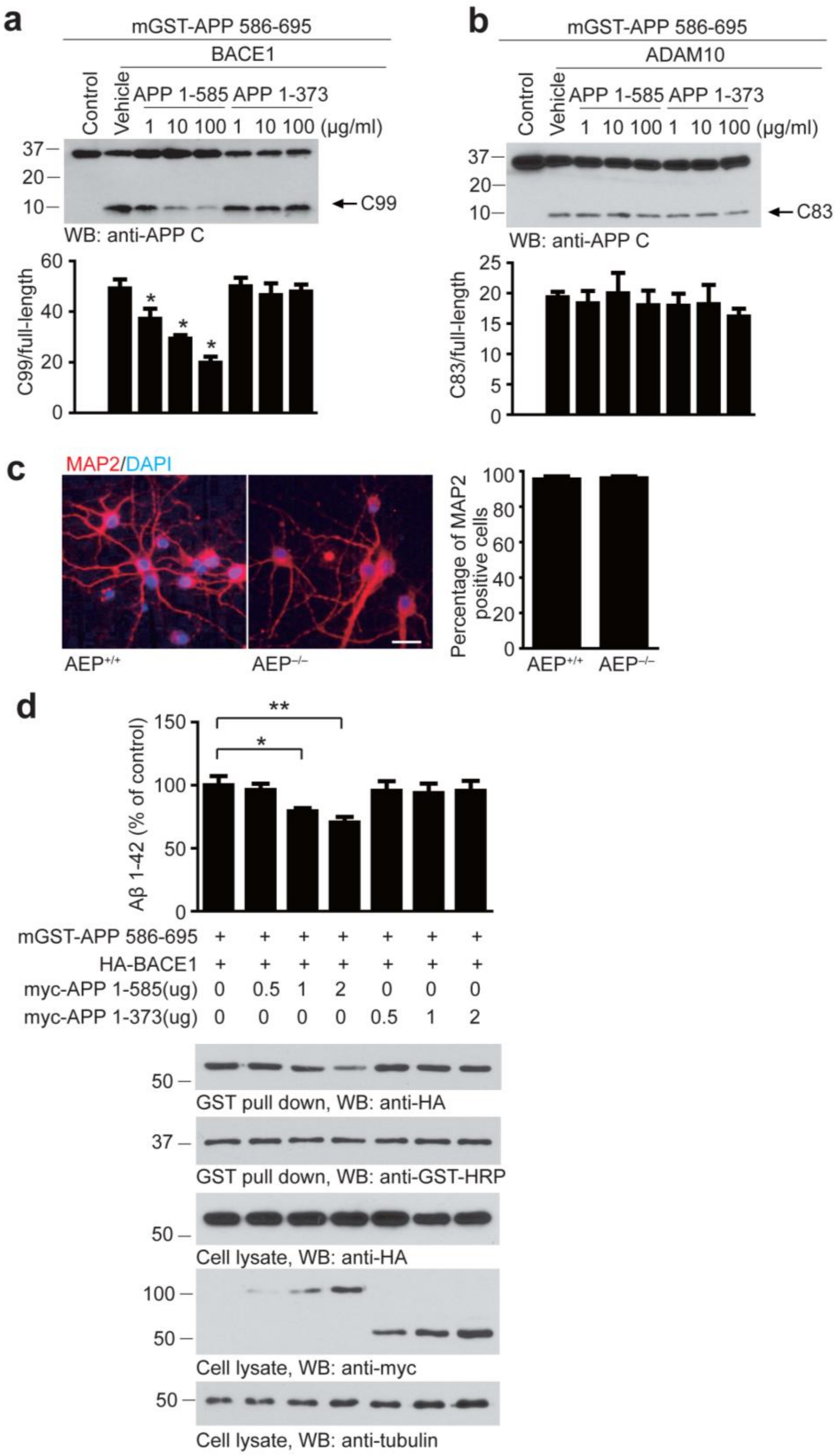

Supplementary Figure 2. APP$_{1-585}$ blocks the cleavage of APP$_{586-695}$ by BACE1. (**a**, **b**) Purified GST-APP$_{586-695}$ recombinant protein was incubated with active BACE1 (**a**) or ADAM10 (**b**) in the presence of increasing amount of recombinant APP$_{1-373}$ or APP$_{1-585}$.

Reactions were analyzed by Western blot using anti-APP C-terminal antibody. Arrows indicate APP C-terminal fragments generated by BACE1 (C99) and ADAM10 (C83) (*P < 0.01 compared with the vehicle group). (**c**) The purity of the cultured primary cortical neurons. The cultured neurons were stained with MAP2 and DAPI. The percentage of MAP2 positive cells was plotted. Scale bar, 20 μm. (**d**) APP fragment$_{1\text{-}585}$ inhibits the binding of BACE1 with APP and the production of Aβ. HEK293 cells were transfected with the indicated constructs. The concentrations of Aβ42 in the medium were analyzed using ELISA (*P < 0.05, ** P < 0.01). The binding of APP fragment (586-695) was analyzed using GST pull-down assay. Data are mean ± s.e.m. of three independent experiments, and were analyzed using t-test (c) or one-way ANOVA (a, b, d) followed by *post hoc* comparison.

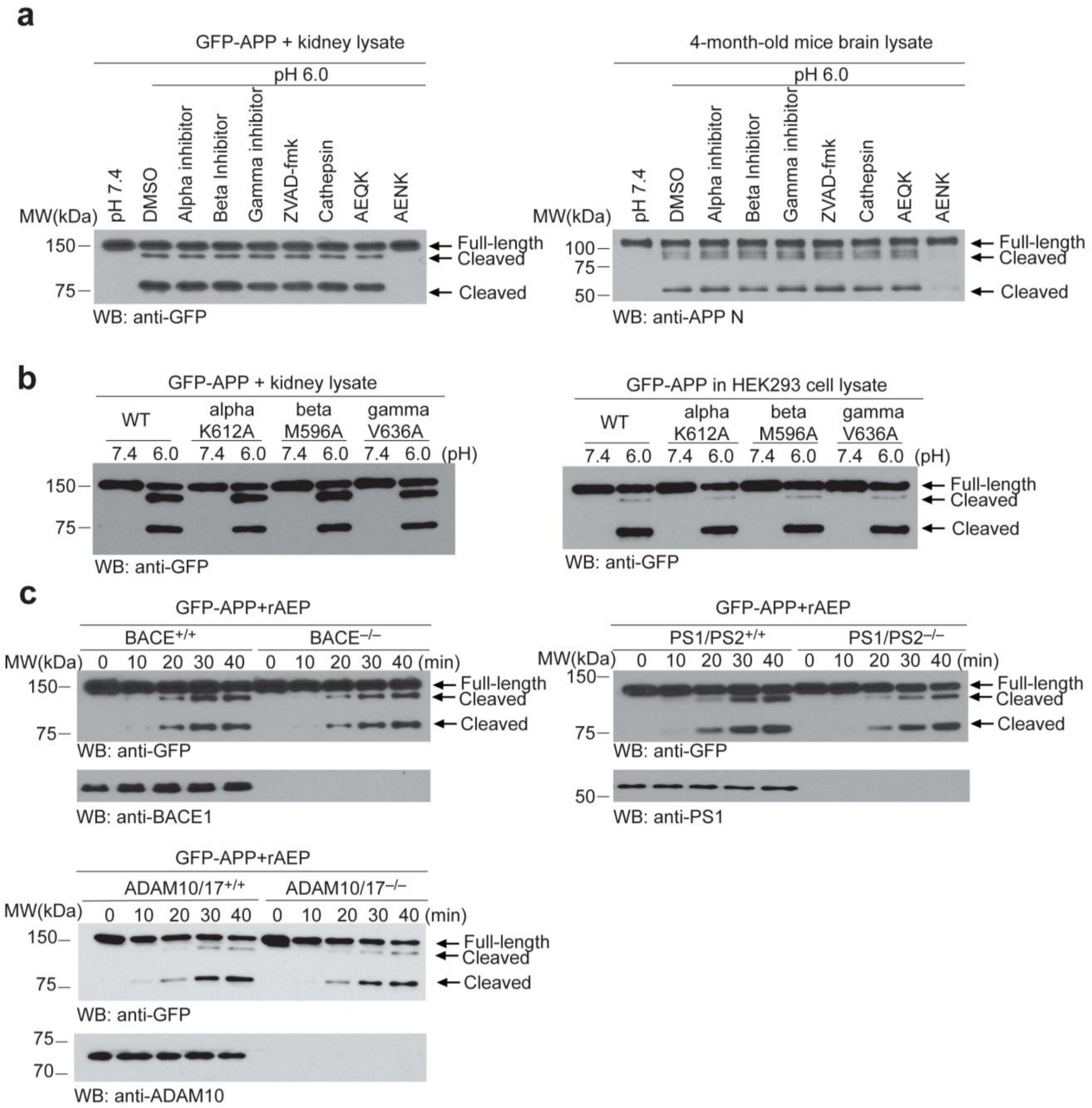

Supplementary Figure 3. The cleavage of APP by AEP is not dependent on secretases. (**a**) Cleavage of APP by AEP is not blocked by secretase, caspase, or cathepsin inhibitors. Left panel, HEK293 cells were transfected with GFP-APP. The cell lysates were incubated with kidney lysates at 37°C for 30 min in the presence or absence of α-secretase inhibitor, β-secretase inhibitor, γ-secretase inhibitor, pan-caspase inhibitor ZVAD-fmk, cathepsin inhibitor E64, AEP peptide inhibitor AENK or control peptide AEQK. Right panel,

4-month-old mouse brain tissues were lysed in pH 6.0 buffer containing the above inhibitors and incubated at 37 ℃ for 30 min. APP cleavage was analyzed by Western blot. (**b**) Cleavage of APP with α-, β-, or γ-secretase cleavage site mutations by AEP. (**c**) The cleavage of APP by AEP is not dependent on α-, β- or γ-secretase activity. WT, BACE1 knockout, PS1/PS knockout, or ADAM10/17 knockout MEF cells were transfected with GFP-APP and lysed in pH 6.0 buffer containing recombinant AEP (5 ug ml^{-1}) for 10, 20, 30, or 40 min, and APP cleavage was analyzed by Western blot.

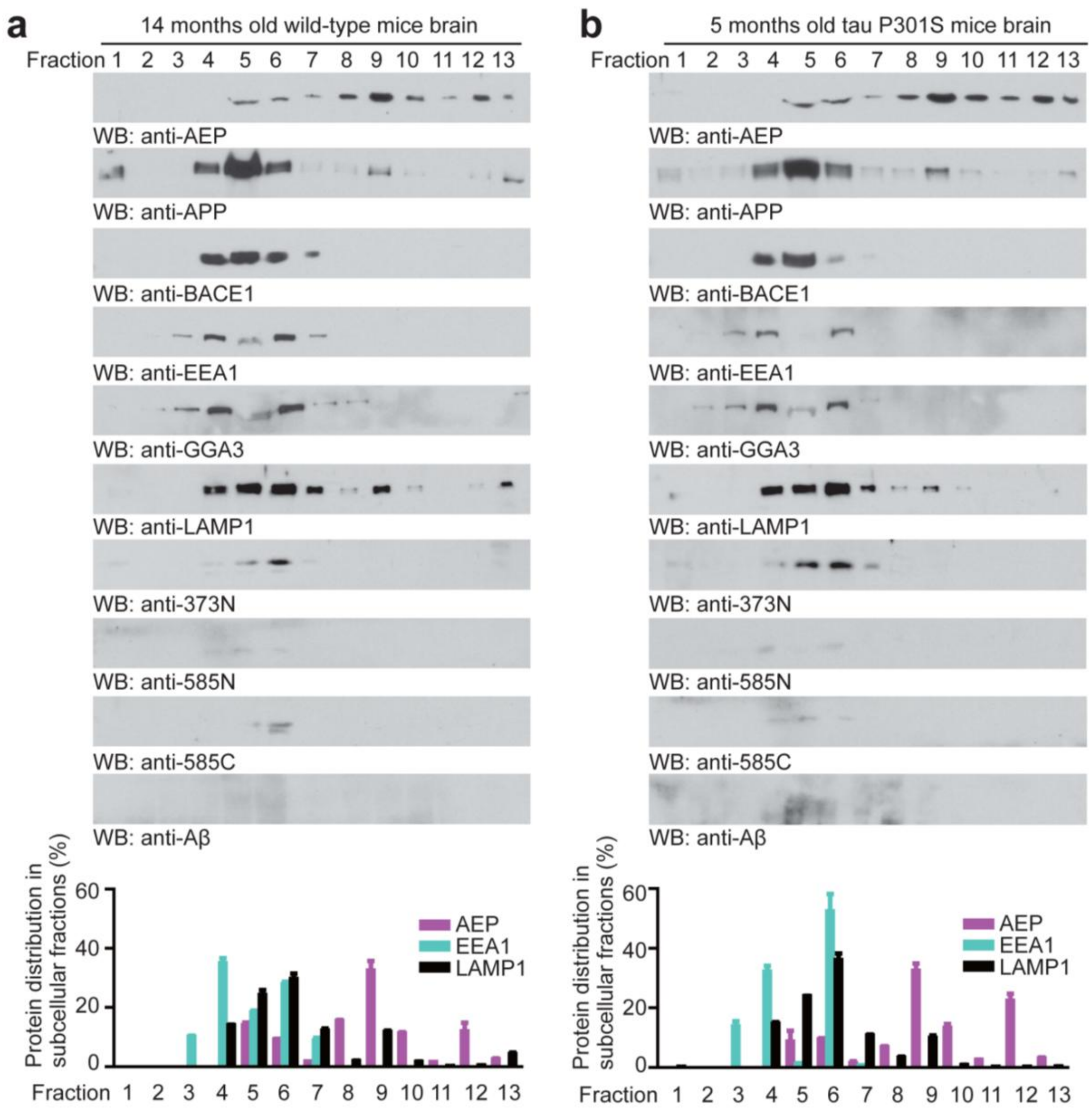

Supplementary Figure 4. AEP and APP distribution in the subcellular fractions of aged mouse brain and tau P301S mouse brain. Brain samples from 14 months old wild-type (**a**) and 5 month-old tau P301S transgenic (**b**) mice were homogenated and fractionated on a discontinuous sucrose gradient. The fractions were analyzed by Western blotting for AEP, APP fragments, BACE1, EEA1 (endosome marker), GGA3 (trans-Golgi network maker), and LAMP1 (lysosome marker). The relative amount of AEP, EEA1, LAMP1 in each fraction were quantified (mean ± s.e.m. of three independent experiments).

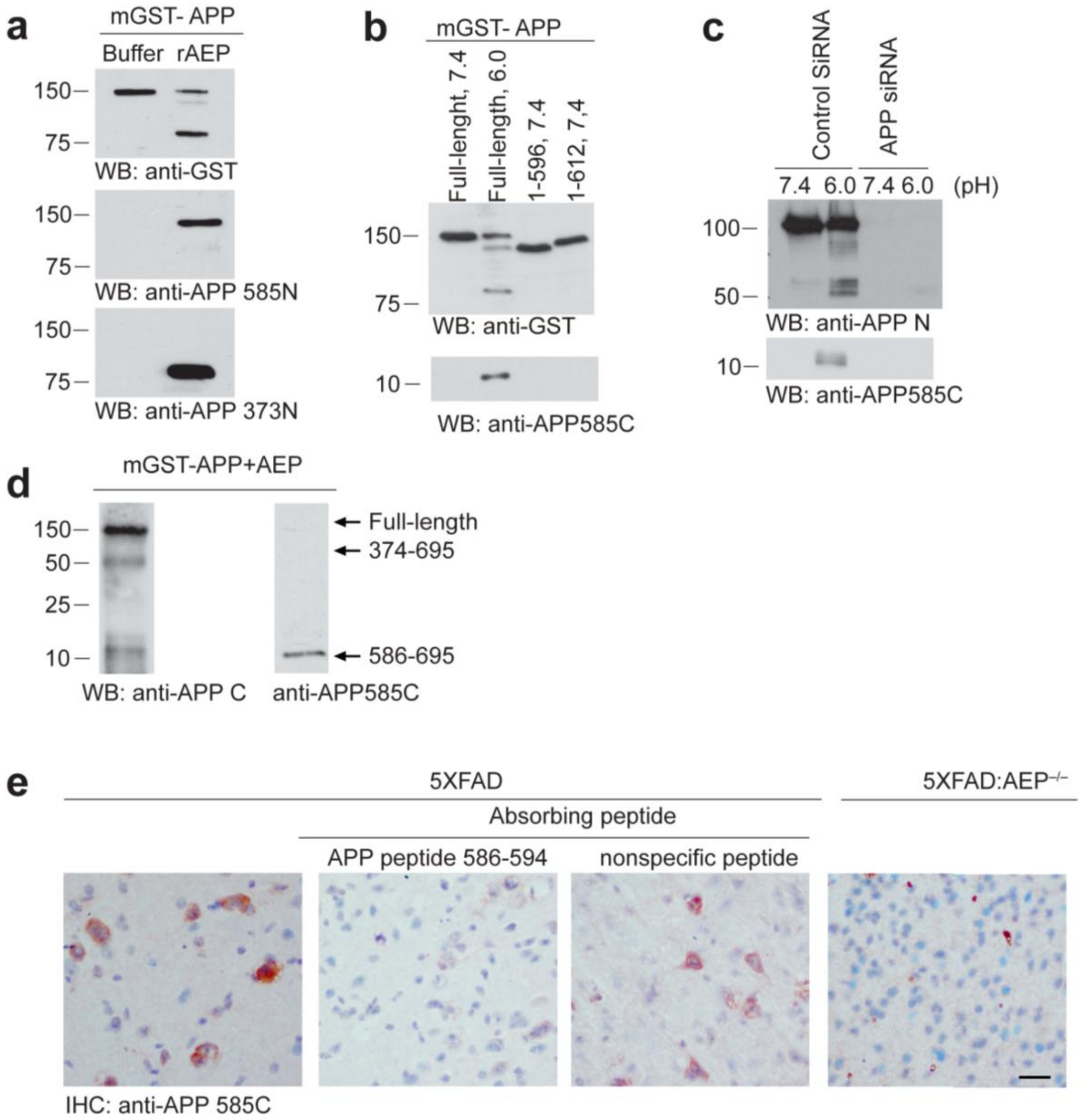

Supplementary Figure 5. Validation of anti-APP 373N, anti-APP 585N, and anti-APP 585C antibodies. (**a**) mGST-APP was incubated with active recombinant AEP. The cleavage of APP was monitored by immunoblotting using anti-GST antibody, anti-APP 585N antibody, and anti-APP 373N antibodies, respectively. The anti-APP 585N and anti-APP 373N antibodies specifically recognized $APP_{1\text{-}373}$ and $APP_{1\text{-}585}$ fragment, respectively, but did not recognize the full-length APP. (**b**) The anti-APP 585C antibody does not recognize the full-length APP, $APP_{1\text{-}596}$, or $APP_{1\text{-}612}$ fragments. (**c**) The specificity of the anti-APP585C antibody was also verified in APP knockdown neurons. (**d**) The AEP-derived APP fragments

were detected by APP C-terminal antibody and anti-APP 585C, respectively. Anti-APP 585 C antibody recognized the $APP_{586-695}$ fragment, but not the full-length APP or $APP_{374-695}$ fragment. (**e**) Detection of AEP-cleaved APP fragments in brain sections of 5XFAD and 5XFAD/$AEP^{-/-}$ mice. Immunohistochemistry using anti-AEP-cleaved APP antibody detected that some of the neurons in the cortex of 5XFAD mice were positive for AEP-cleaved APP. The signal was blocked by $APP_{586-594}$ peptide, but not by the nonspecific peptide. The positive signal was dramatically diminished in 5XFAD/$AEP^{-/-}$ mouse brain. Scale bar, 50 μm.

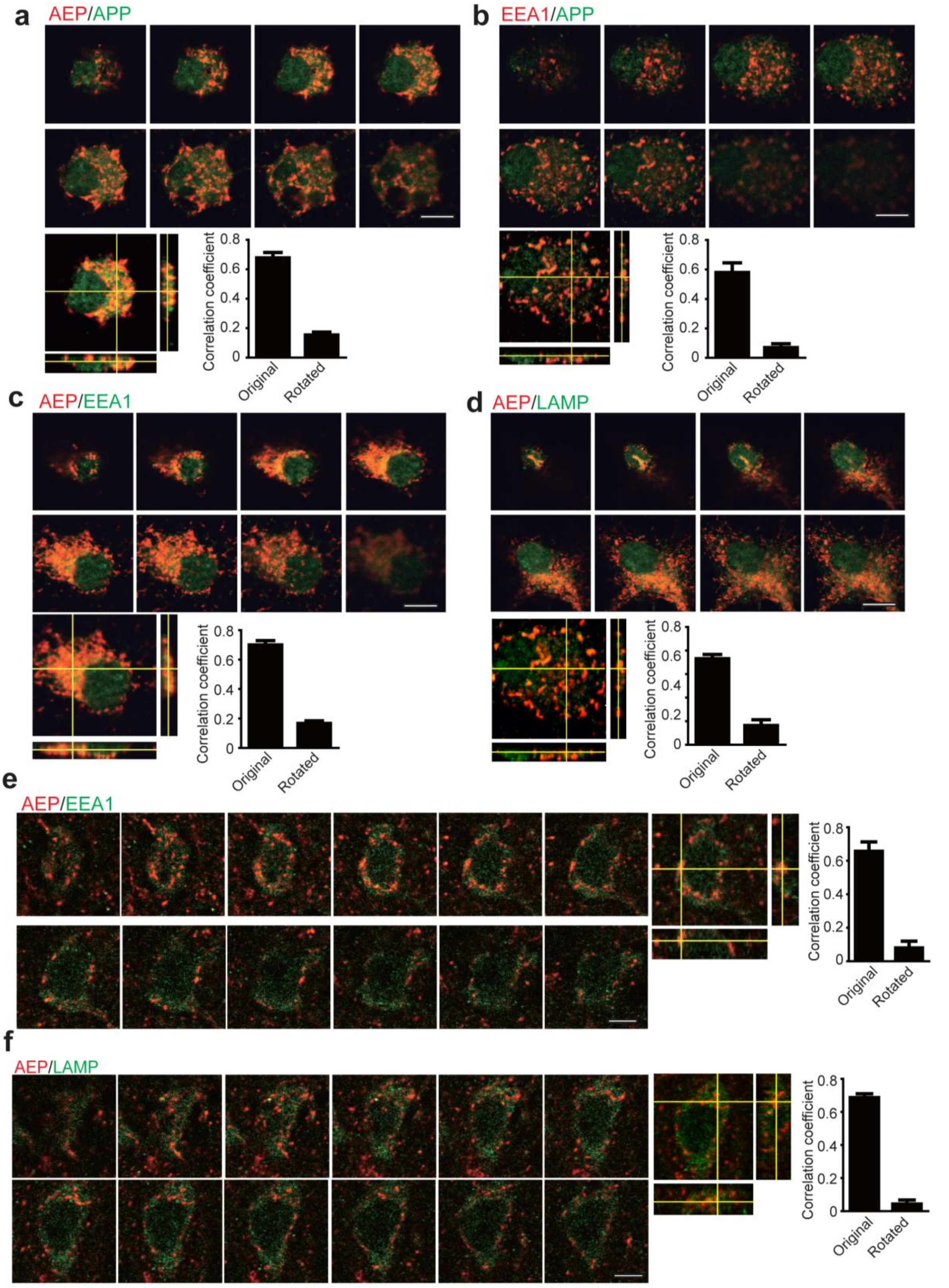

Supplementary Figure 6. Localization of AEP in endosomes and lysosomes. (**a**, **b**) Z-stack confocal microscopy images of AEP/APP and EEA1/APP colocalization after the neurons were treated with 20 μM pre-aggregated Aβ for 24 h. (**c**, **d**) Z-stack confocal

microscopy images showing AEP colocalizes with EEA1 and LAMP1 in primary neurons treated with 20 μM of pre-aggregated Aβ for 24 h. (**e**, **f**) Z-stack confocal microscopy images showing AEP colocalizes with EEA1 and LAMP1 in 5XFAD mice brain. Shown are the representative figures of two independent experiments. Scale bar, 10 μm. The bar graphs show the Pearson's correlation coefficient (PCCs) of the red and green images before and after rotating the red image by 90 degrees. Before rotation, the red images associate with the green image. After rotation, only random colocalization is observed.

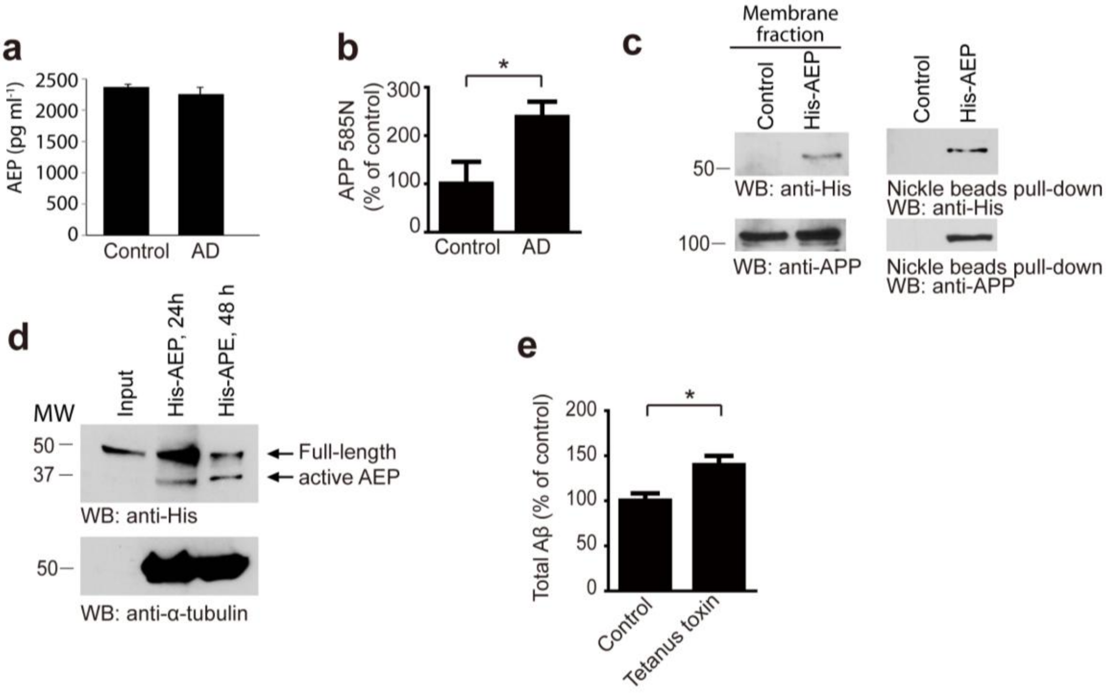

Supplementary Figure 7. AEP binds to APP on the cell surface, and is internalized and processed to its active form. (**a**) ELISA determination of the AEP levels in the cerebral spinal fluid (CSF) of control and AD patients (mean ± s.e.m.). (**b**) Protein level of $APP_{1\text{-}585}$ fragment in the CSF of control and AD patients. Human control and AD CSF samples were analyzed by Western blot using APP585N antibody. The densitometry of western blot bands was quantified (mean ± s.e.m.; *t* test, **P* < 0.01). (**c**) AEP binds to APP on the cell surface. Exogenous His-tagged AEP was added to the medium of cultured neurons. 24 h later, the His-AEP in the membrane fraction was pull down with nickel beads. APP was coprecipitated with AEP. (**d**) Exogenous His-tagged AEP was internalized and processed to active form. His-tagged AEP was added in to the medium of cultured neurons. 24 h and 48 h later, Western blot show that AEP was processed into its active form. (**e**) ELISA analysis of total Aβ level in the cell lysate of HEK293 cells stably transfected with APP in the presence or absence of 20 nM tetanus toxin (mean ± s.e.m.; *t*-test, **P* < 0.01).

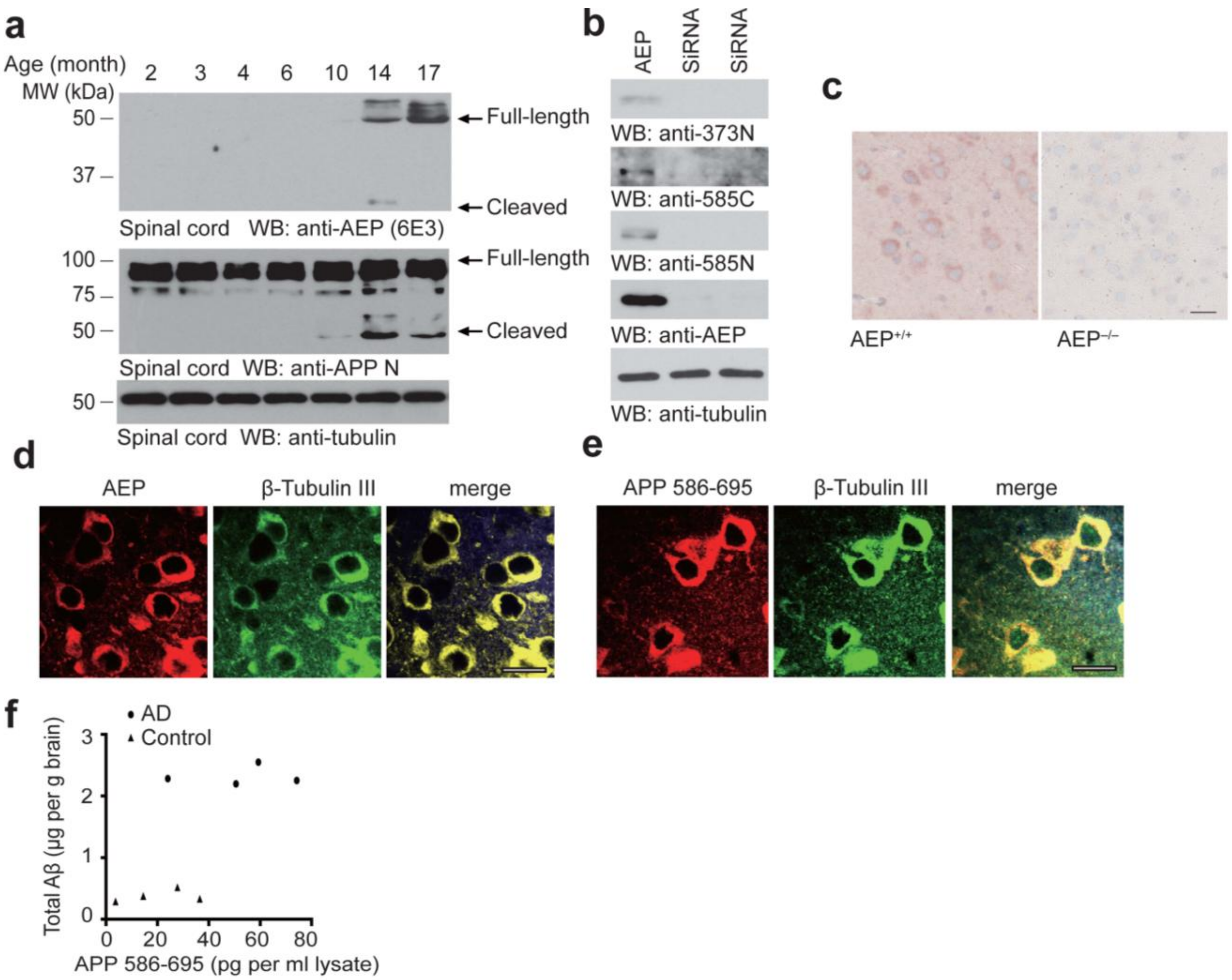

Supplementary Figure 8. Cleavage of APP by AEP in AD central nervous system. (**a**) Spinal cord lysates from 2-, 3-, 4-, 6-, 10-, 14-, and 17-month-old mice were analyzed by Western blot. (**b**) Cleavage of endogenous APP by AEP in HEK293 cells. AEP-derived fragments were detected in HEK293 cells. AEP siRNA blocked the production of these fragments. (**c**) AEP expression in wild-type and $AEP^{-/-}$ mouse brain. (**d**) Confocal analysis showing AEP colocalizes with neuronal marker β-Tubulin III 5XFAD mice brain. (**e**) Confocal analysis showing $APP_{586\text{-}695}$ fragment colocalizes with neuronal marker β-Tubulin III 5XFAD mice brain. Scale bar, 20 μm. (**f**) Correlation between AEP-derived APP fragment and Aβ in AD and age-matched control brain tissue.

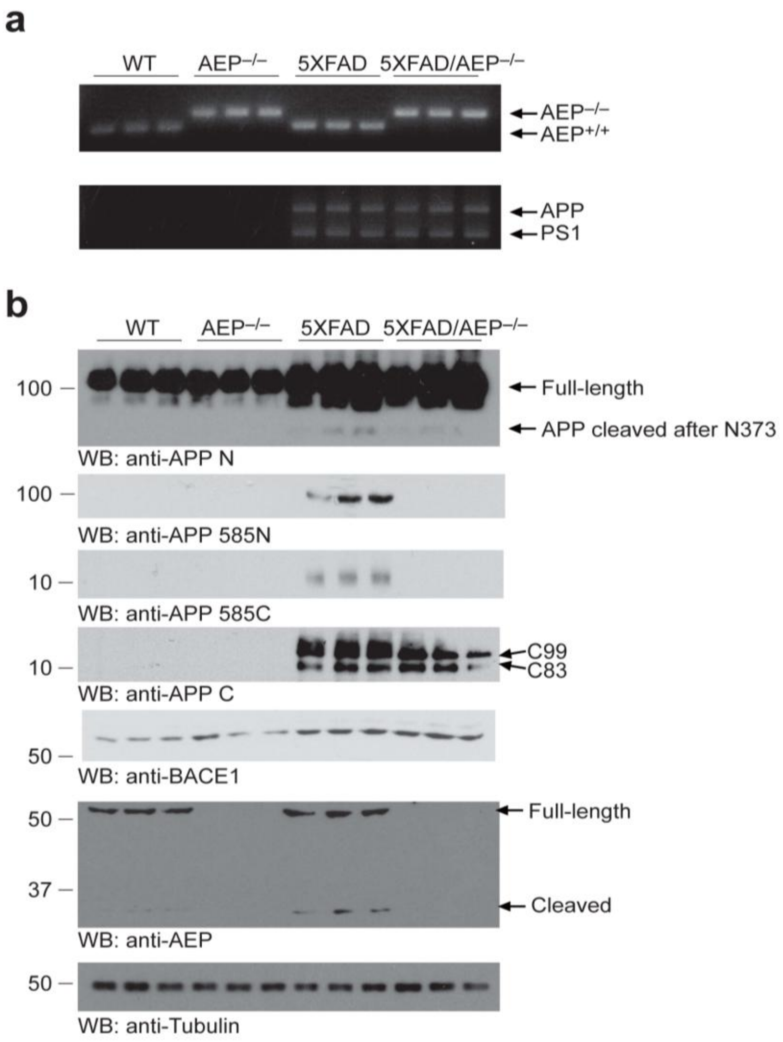

Supplementary Figure 9. Characterization of 5XFAD/AEP$^{-/-}$ mice. (**a**) Genotyping of wild-type, AEP$^{-/-}$, 5XFAD and 5XFAD/AEP$^{-/-}$ mice. (**b**) Validation of the protein expression and processing of APP. Immunoblotting using anti-APP N-terminal antibody detected that the expression of APP in 5XFAD mice was much higher than that in wild type and AEP$^{-/-}$ mice. AEP knockout did not interfere with the expression of APP. The AEP-derived APP fragments were found in 5XFAD mouse brain but not 5XFAD/AEP$^{-/-}$ mouse brain. Immunoblotting using anti-APP C-terminal antibody found that C99 was decreased in 5XFAD/AEP$^{-/-}$ mice when compared with 5XFAD mice, while C83 was not affected by AEP deletion.

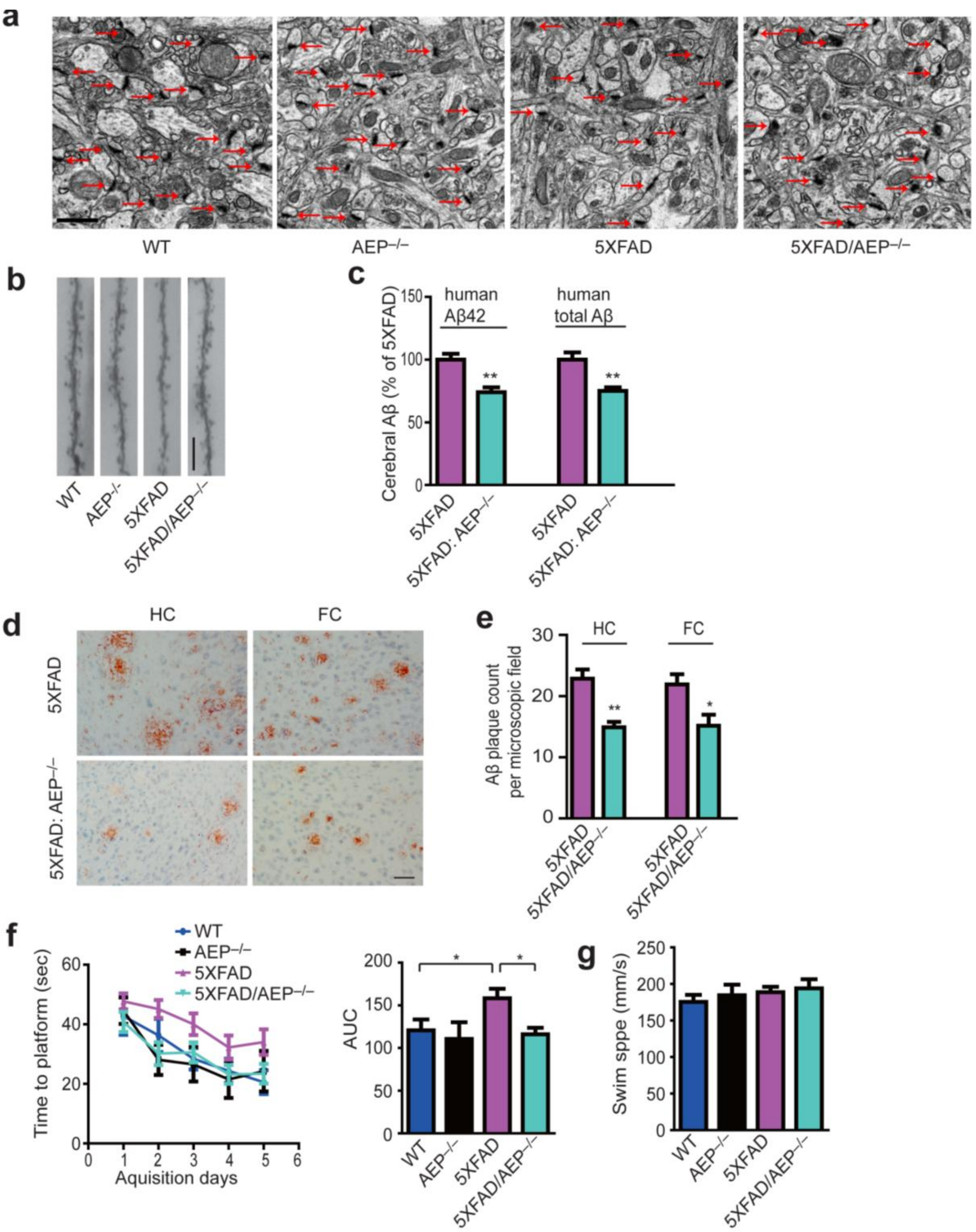

Supplementary Figure 10. AEP gene deficiency protects 5XFAD mice from synaptic loss and special memory deficits. (**a**) Representative electron microscopy images of the synapse. Arrows indicate synapse. Scale bar, 1 μm. (**b**) Golgi staining revealed the dendritic spines from apical dendritic layer of the CA1 region. Scale bar, 5 μm. (**c**) ELISA quantification of

Aβ in the brain of 1.5-month-old 5XFAD and 5XFAD/AEP$^{-/-}$ mice (mean ± s.e.m.; n=9 in 5XFAD, n=10 in 5XFAD/AEP$^{-/-}$ mice. **P < 0.01). (**d**) Immunostaining of Aβ deposits in the hippocampus (HC) and frontal cortex (FC). Scale bar, 50 uM. (**e**) Quantification of number of Aβ plaques (mean ± s.e.m.; n = 6 in each group; *P < 0.05, **P < 0.01). (**f**) Morris water maze analysis as latency traveled (second) and integrated latency (AUC) for WT (n=8), AEP$^{-/-}$ (n=8), 5XFAD (n=9) and 5XFAD/AEP$^{-/-}$ (n=10) mice (mean ± s.e.m.; *P < 0.05). (**g**) AEP deletion does not interfere with mice swim speed. Swim speed during the Morris water maze test were recorded and analyzed, statistical analysis did not reveal any significant differences between groups (mean ± s.e.m.). Data were analyzed using *t*-test (c, e) or one-way ANOVA followed by *post hoc* comparison (f, g).

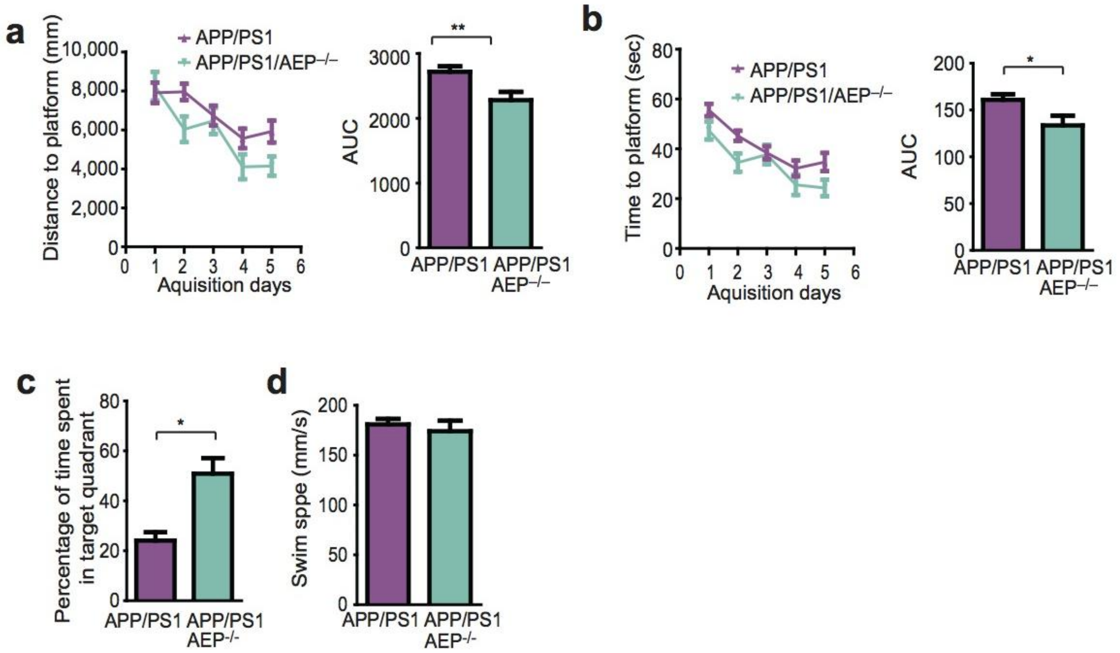

Supplementary Figure 11. AEP gene deficiency prevents APP/PS1 mice from special memory deficits. Spatial memory of 15-month-old APP/PS1 and APP/PS1/AEP$^{-/-}$ mice were tested by Morris water maze test. (**a**) Distance travelled (millimeters) and integrated distance (AUC) for APP/PS1 (n=15) and APP/PS1/AEP$^{-/-}$ (n=13) mice (mean ± s.e.m.; $^{**}P < 0.01$). (**b**) Time needed to reach the hidden platform (second) and integrated time (AUC) for APP/PS1 (n=15) and APP/PS1/AEP$^{-/-}$ (n=13) mice (mean ± s.e.m.; $^{*}P < 0.05$). (**c**) Probe trial result. Shown is the mean ± s.e.m. percentage of time spent in the target quadrant ($^{*}P < 0.05$). **(d)** AEP deletion does not interfere with mice swim speed (mean ± s.e.m.). Data were analyzed using *t*-test.

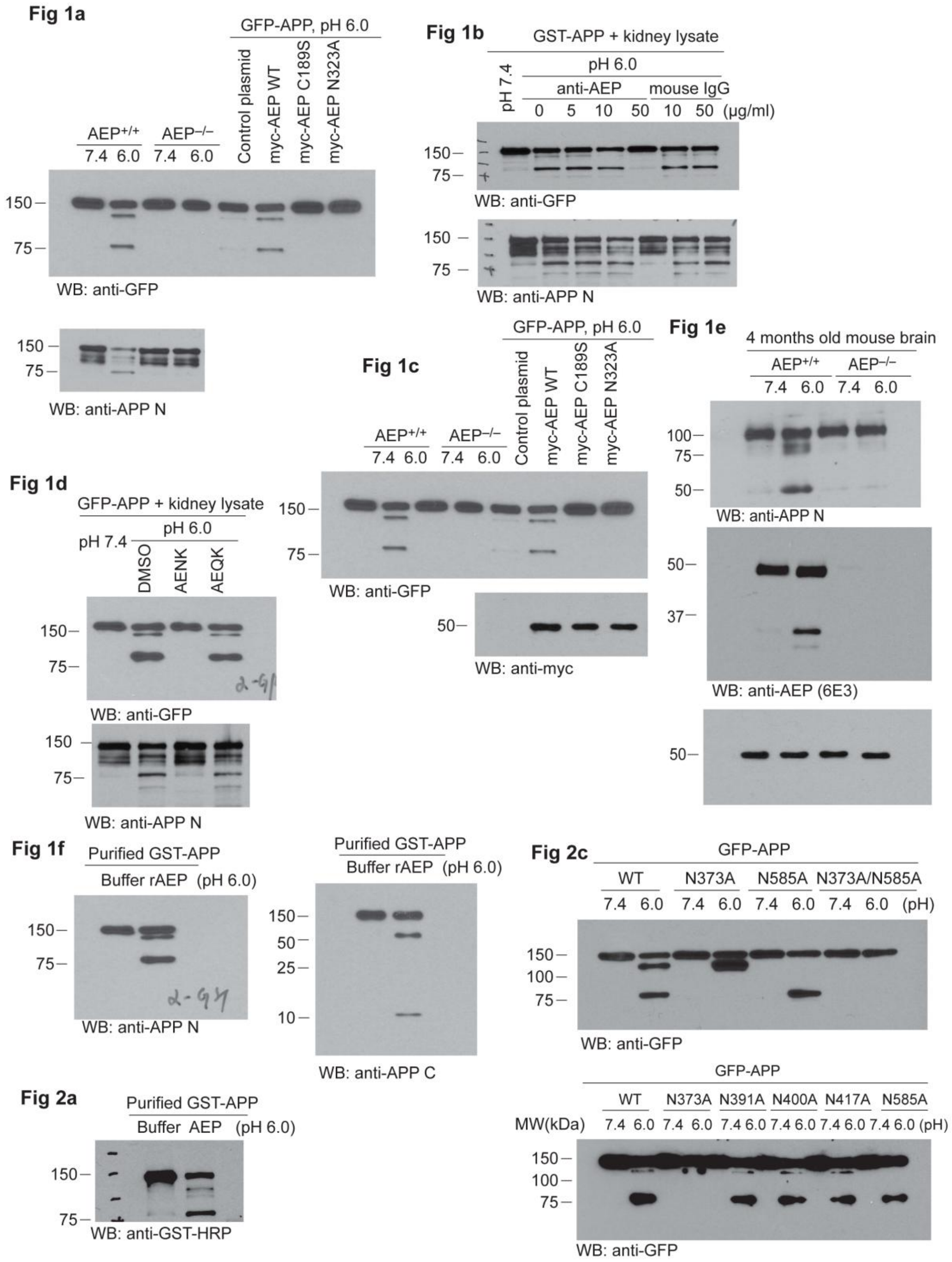

Supplementary Figure 12. Original scans of Western blots.

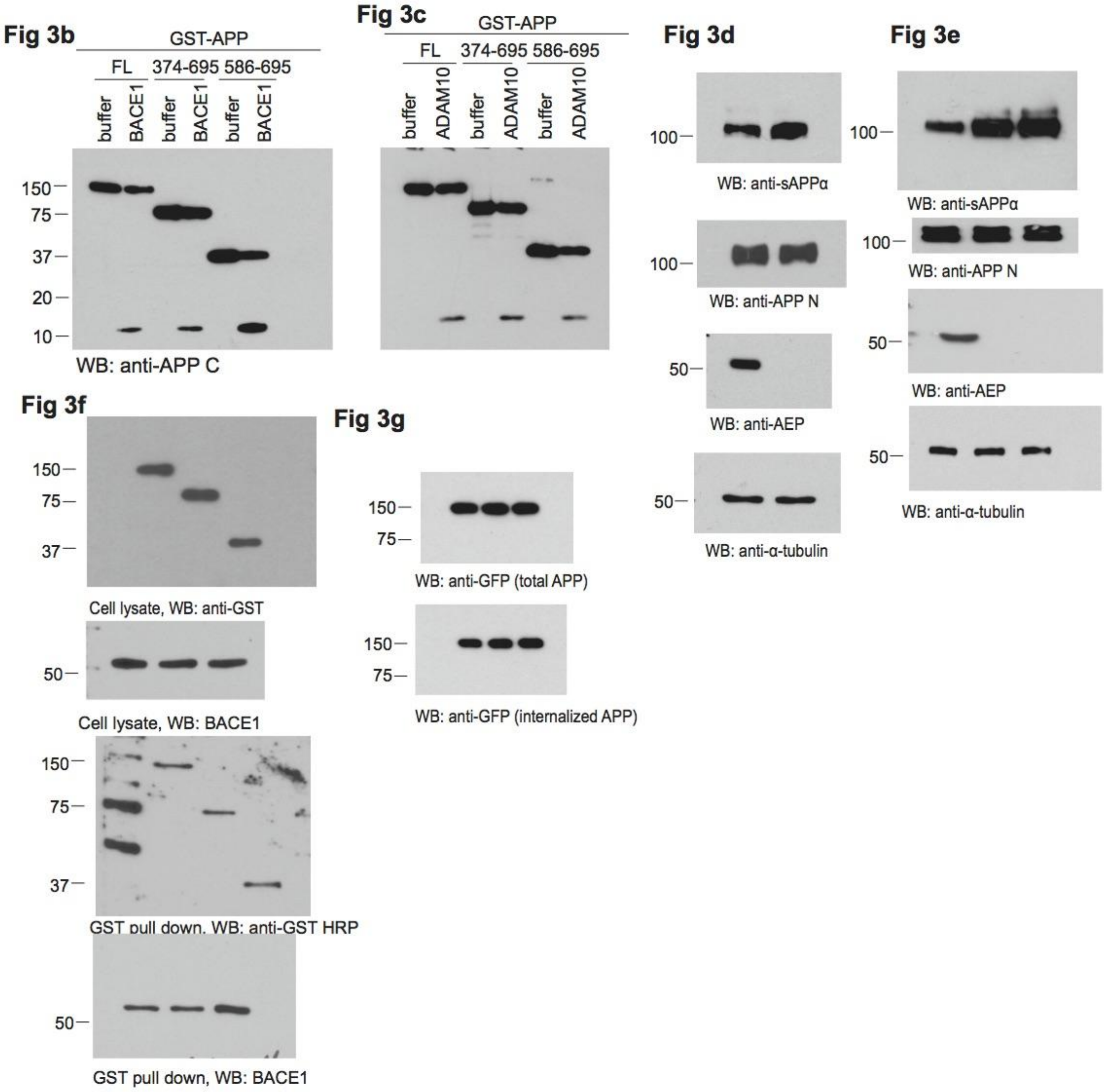

Supplementary Figure 12. Original scans of Western blots (con).

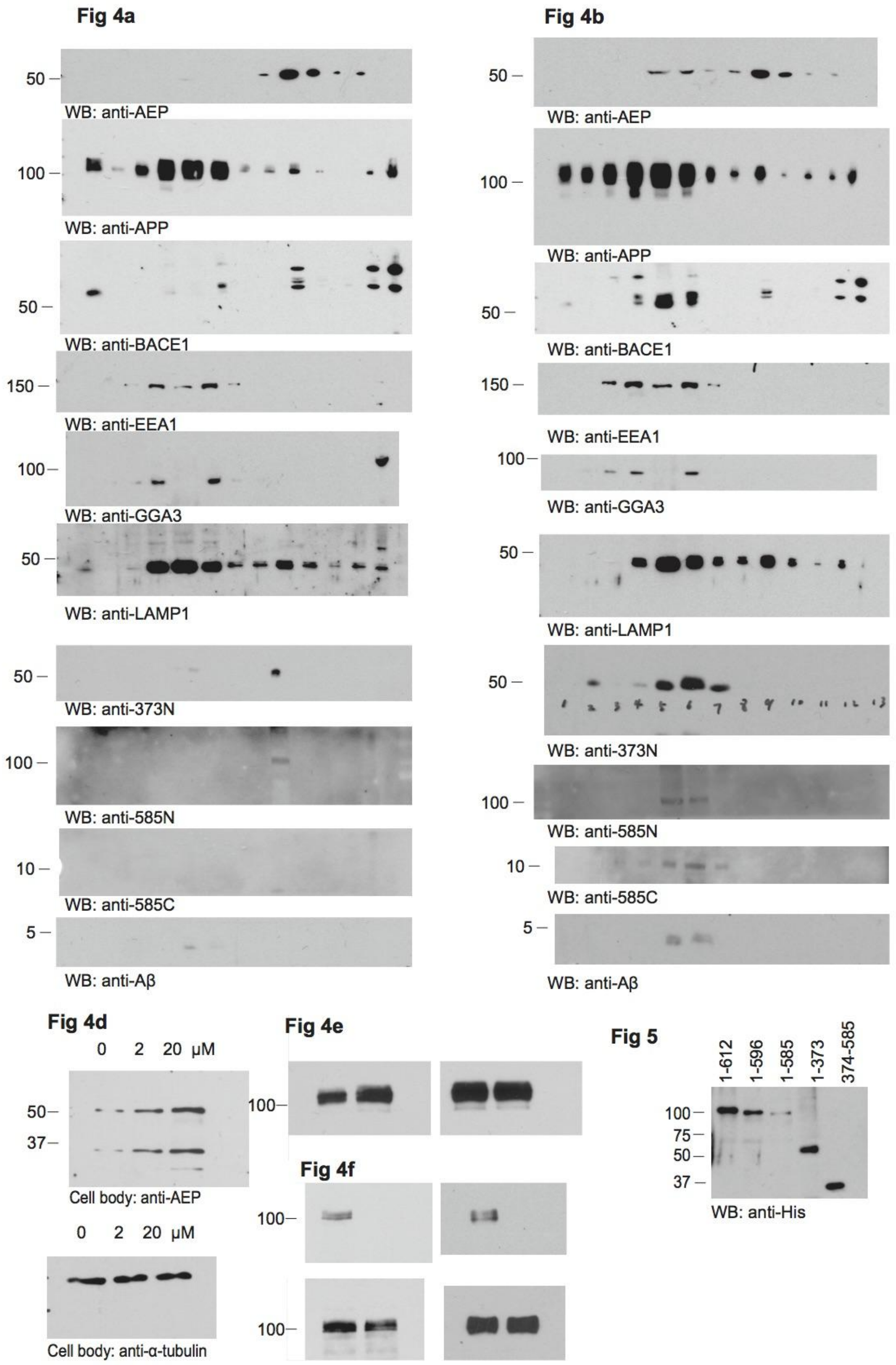

Supplementary Figure 12. Original scans of Western blots (con).

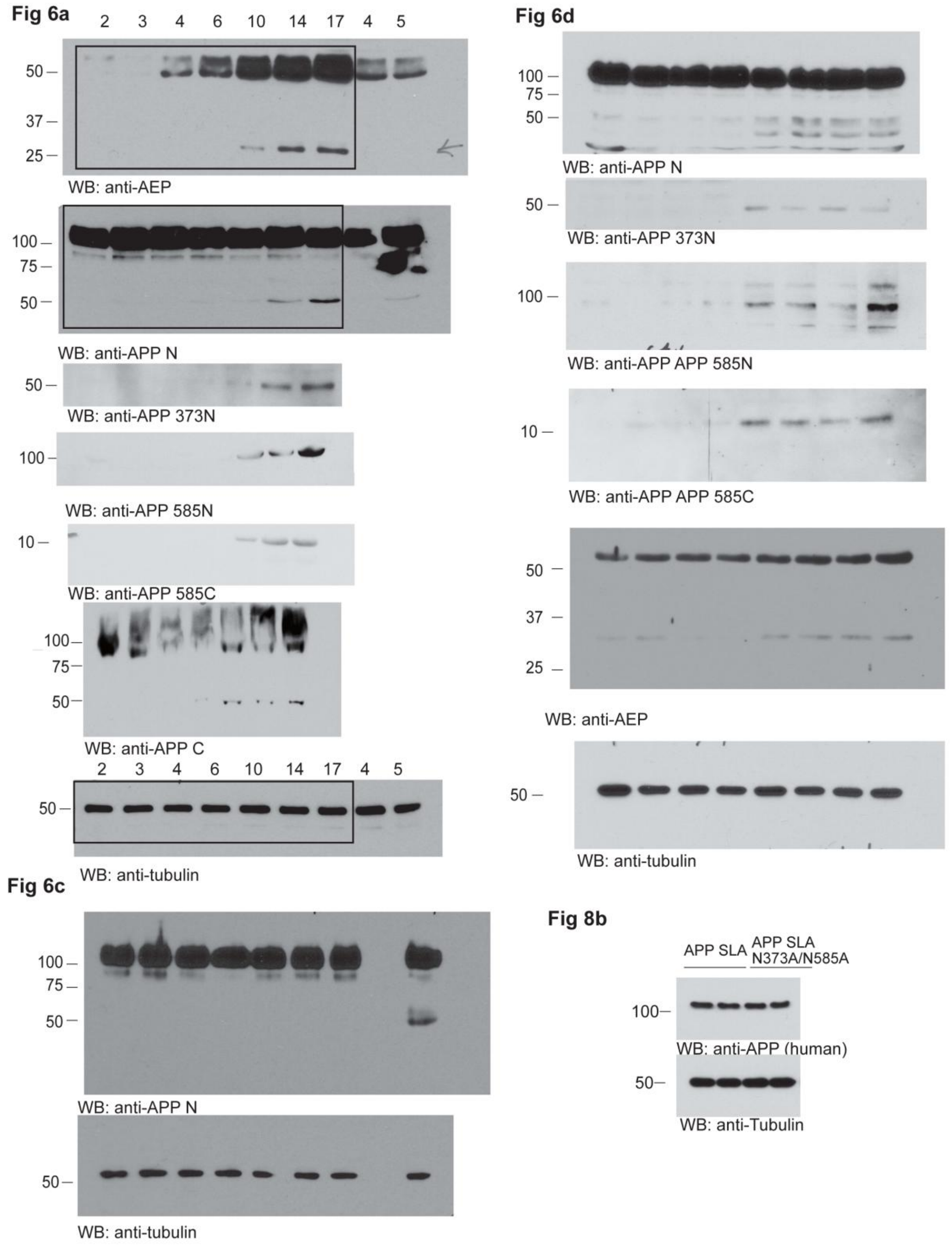

Supplementary Figure 12. Original scans of Western blots (con).

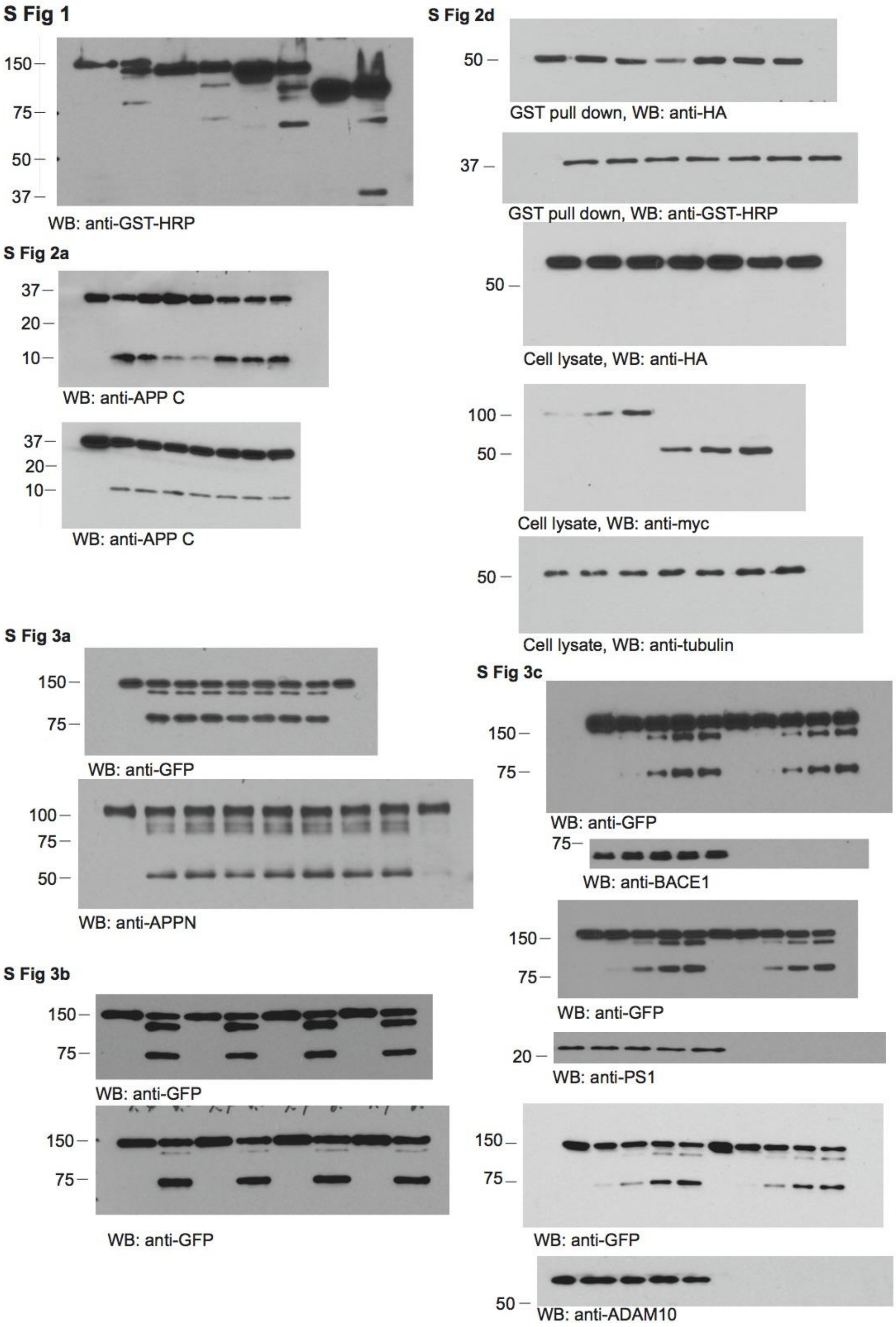

Supplementary Figure 12. Original scans of Western blots (con).

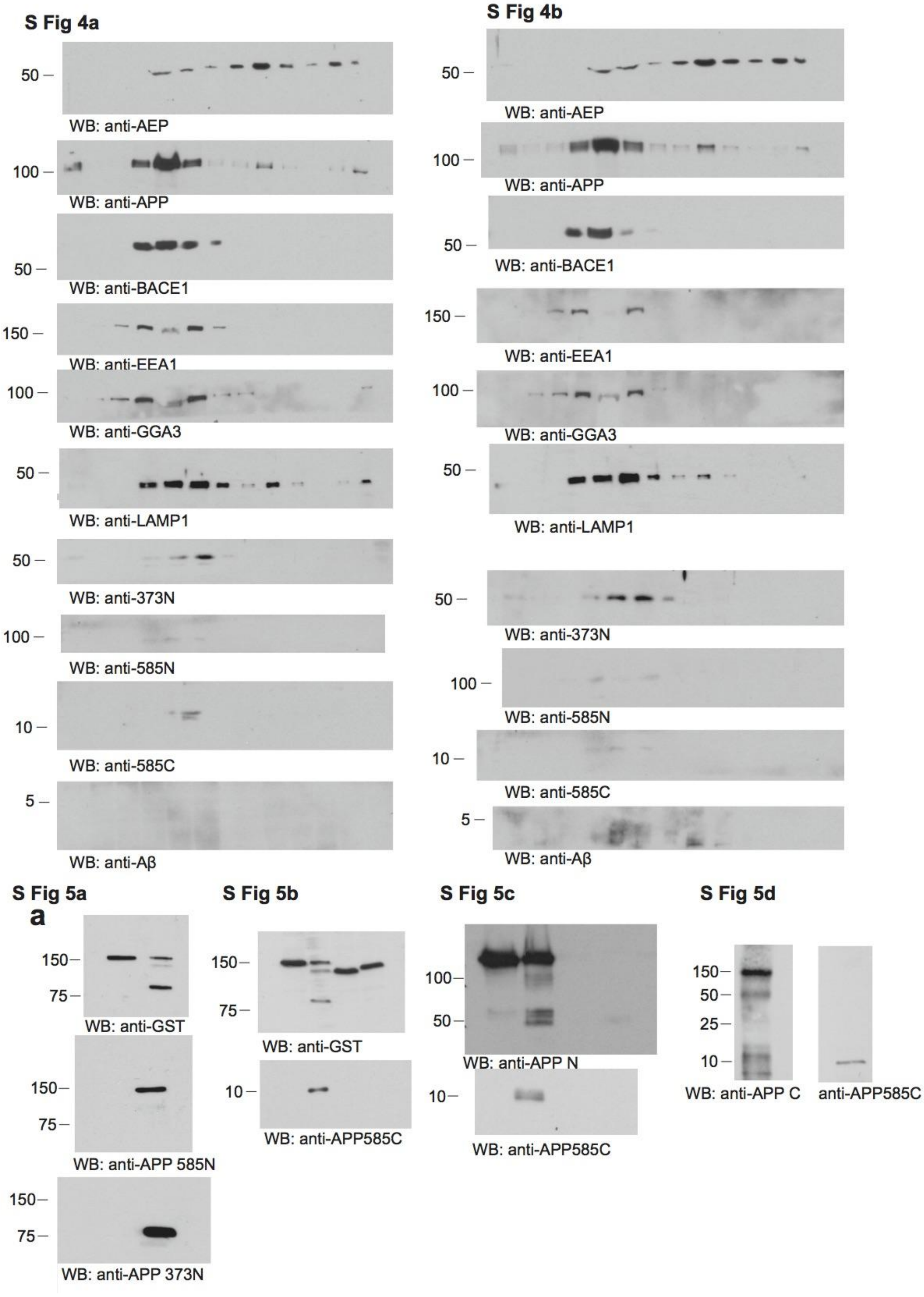

Supplementary Figure 12. Original scans of Western blots (con).

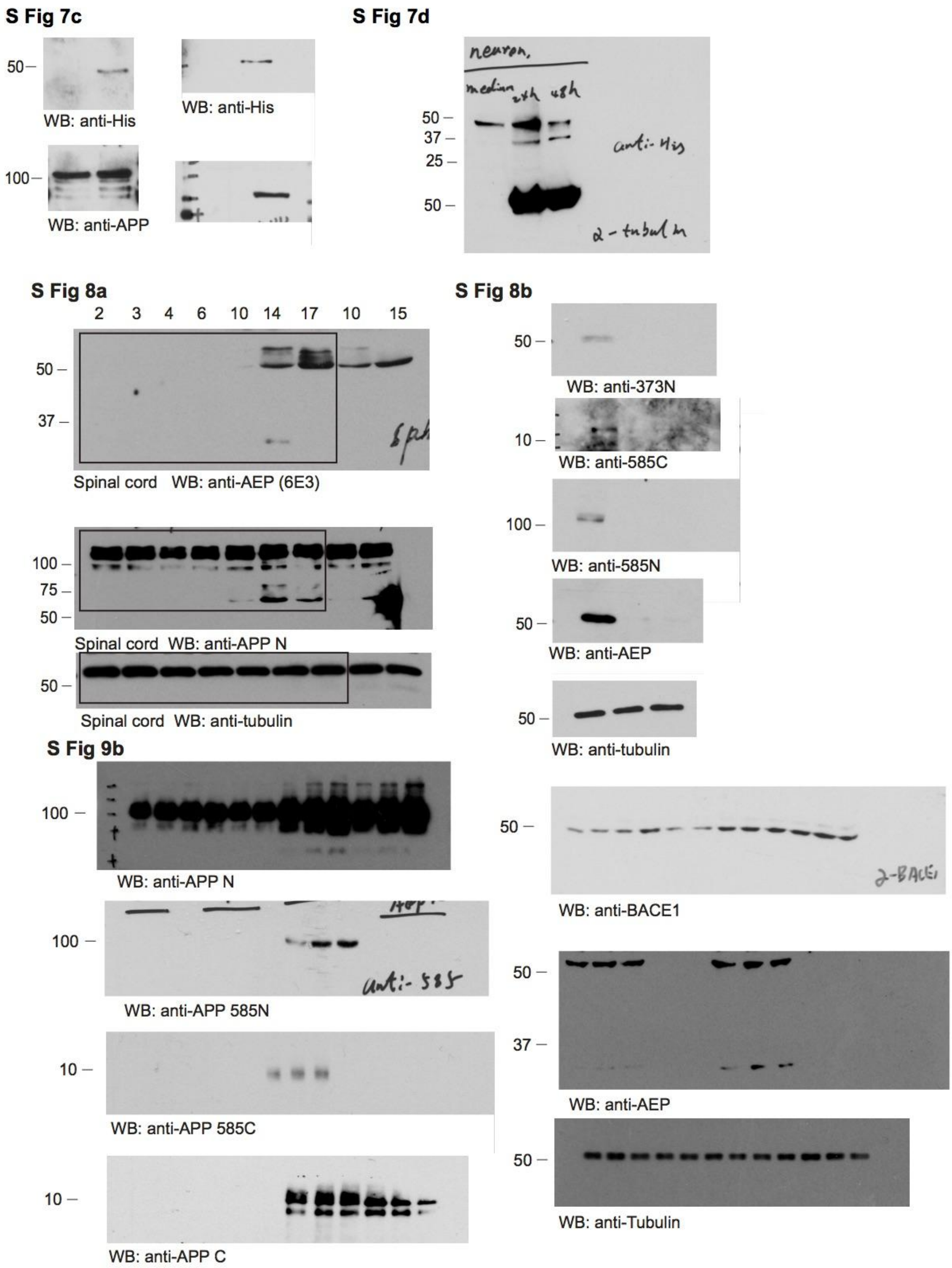

Supplementary Figure 12. Original scans of Western blots (con).
